# Supplementary material for: A Global Ligandability Map of Tryptoline Butynamide Stereoprobes Identifies Covalent Inhibitors of the Actin Maturation Protease
Source: J Am Chem Soc. 2026 May 20;148(21):22077–90. doi: 10.1021/jacs.6c03985 (PMC13367750; doi:10.1021/jacs.6c03985)
Supplement: Supplementary file 2 [file ja6c03985_si_002.pdf]

A Global Ligandability Map of Tryptoline Butynamide Stereoprobes Identifies Covalent Inhibitors of the Actin Maturation Protease

Yijun Xiong[1], Christopher J. Reinhardt[1], Tracey Nguyen[2], Melissa Hoffman[2], Gabriel M. Simon[2], Bruno Melillo[1*], Benjamin F. Cravatt[1*]

1.Department of Chemistry, Scripps Research, La Jolla, California 92037, United States

2.Vividion Therapeutics, San Diego, California 92121, United States

*Correspondence –bmelillo@scripps.edu, cravatt@scripps.edu

Table of Contents

**Supplementary Table and Figures**	**S3**
**Reagents and Materials**	**S14**
**Experimental Methods**	**S16**
**Synthesis and Characterization of Novel Compounds**	**S24**
**References**	**S40**

Supplementary Table and Figures

Tryptoline acrylamides	$M^{-1}s^{-1}$	Time (h)	Average $M^{-1}s^{-1}$	Tryptoline butynamides	$M^{-1}s^{-1}$	Time (h)	Average $M^{-1}s^{-1}$	Stereo-configuration
WX-02-16	0.09	6	0.09	WX-02-621	0.023	6	0.023	1*S*,3*S*
	0.09	2			ND	2		
WX-02-26	0.12	6	0.13	WX-02-623	0.043	6	0.057	1*R*,3*S*
	0.13	2			0.071	2		
WX-02-36	0.08	6	0.08	WX-02-622	0.026	6	0.028	1*R*,3*R*
	0.08	2			0.029	2		
WX-02-46	0.10	6	0.10	WX-02-624	0.05	6	0.054	1*S*,3*R*
	0.10	2			0.058	2		

Table S1. Glutathione reactivity values for tryptoline acrylamide and butynamide stereoprobes. Rates ($M^{-1}\cdot s^{-1}$) of glutathione consumption by stereoprobes at 2 and 6 h. ND, not determined. Acrylamide data are from a previous publication.[1]

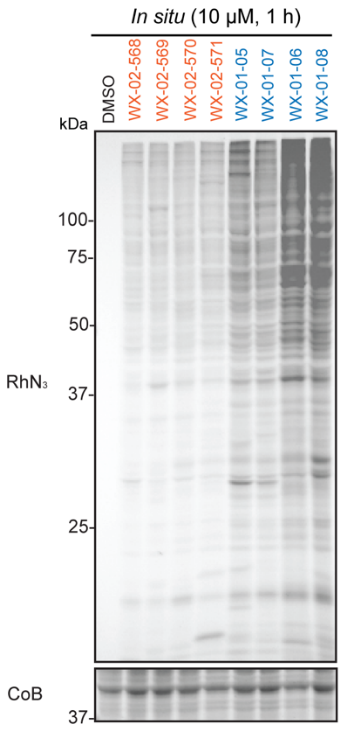

Figure S1. Gel-ABPP data for Ramos cells treated with alkyne stereoprobes (10 µM) for 1 h, followed by lysis and visualization of stereoprobe-reactive proteins by copper-catalyzed azide-alkyne cycloaddition (CuAAC) conjugation[2, 3] to an azide-rhodamine (RhN_3) reporter group, SDS-PAGE, and in gel fluorescence scanning.[4] CoB, Coomassie blue staining. The names of tryptoline butynamides and tryptoline acrylamides are shown in orange and blue font, respectively. Data correspond to the gel-ABPP experiment shown in **Figure 1B** (exposure time 240 s), here with shorter exposure time (120 s).

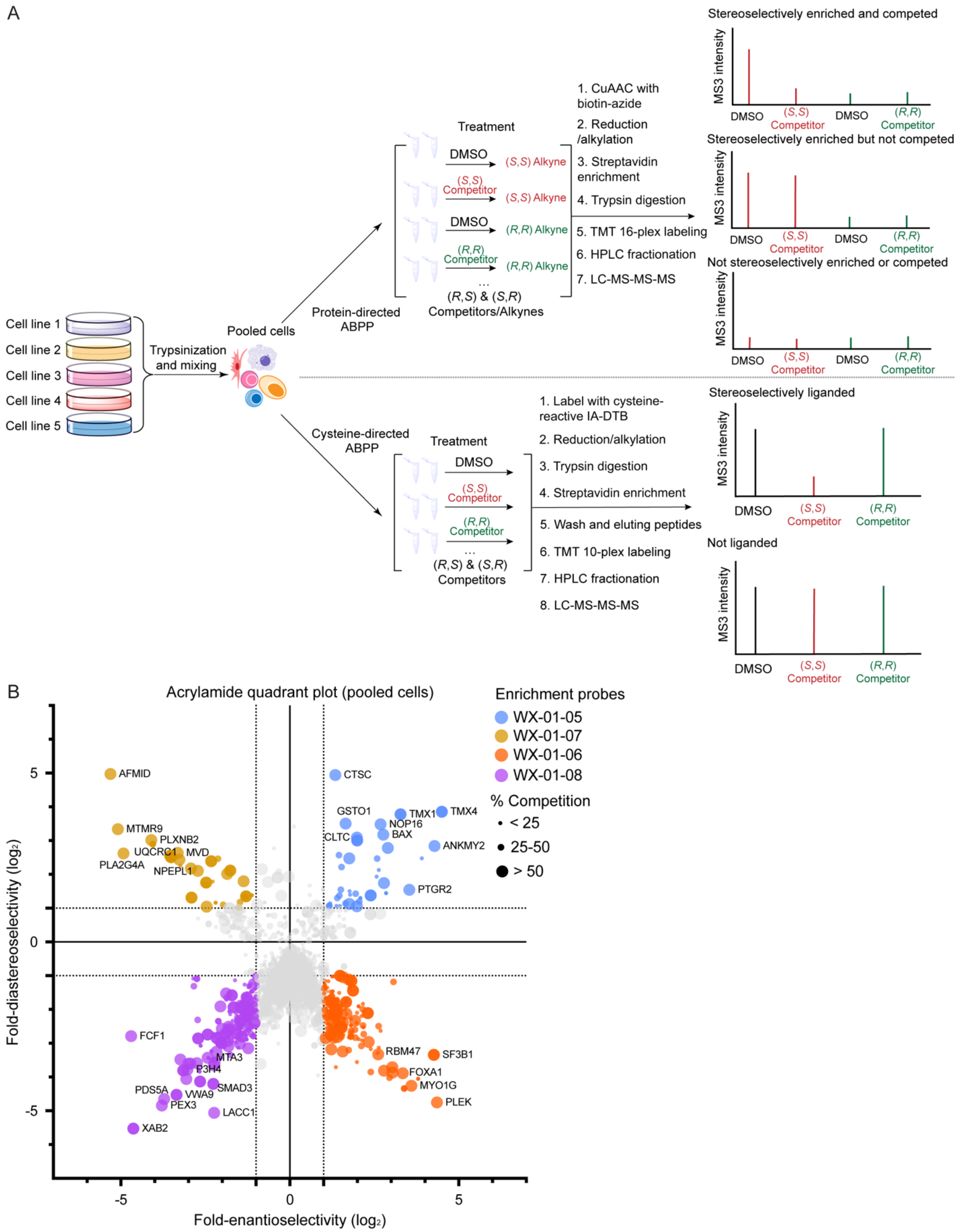

Figure S2. Mapping proteins preferentially liganded by tryptoline butynamides and acrylamides by protein-directed ABPP. (A) General workflow for protein-directed and cysteine-directed ABPP: Ramos, 22Rv1, UACC-257, U-2 OS, and Hep G2 were cultured separately and pooled on the day

of treatment based on equal protein content predetermined by bicinchoninic acid assay (BCA) assay. All cell treatments were performed in a pooled suspension state. Protein-directed ABPP: pretreatment with DMSO or parent competitor stereoprobes (20 µM) for 2 h, followed by stereomatched alkyne stereoprobe (5 µM) for 1 h; stereoselective enrichment of proteins by alkyne stereoprobes and blockade of enrichment by stereomatched non-alkyne competitor stereoprobes were determined by multiplexed (tandem mass tagging, TMT^{16plex}) MS-based proteomics.[1, 5] Cysteine-directed ABPP: treatment with parent stereoprobes (20 µM) for 3 h; stereoprobe reactivity with cysteines was determined by multiplexed (TMT^{10plex}) MS-based proteomics.[6] Mock data on the right show examples of liganded proteins (protein-directed ABPP) and cysteines (cysteine-directed ABPP). (B) Quadrant plots highlighting stereoselectively liganded proteins for each stereoconfiguration of tryptoline acrylamides as determined by protein-directed ABPP. Enantioselectivity (*x*-axis) is the ratio of enrichment for one stereoisomer versus its enantiomer, and diastereoselectivity (*y*-axis) is the ratio of enrichment of one stereoisomer versus the average of its two diastereomers. For B, the protein-directed ABPP data represent average values from four independent experiments per stereoprobe.

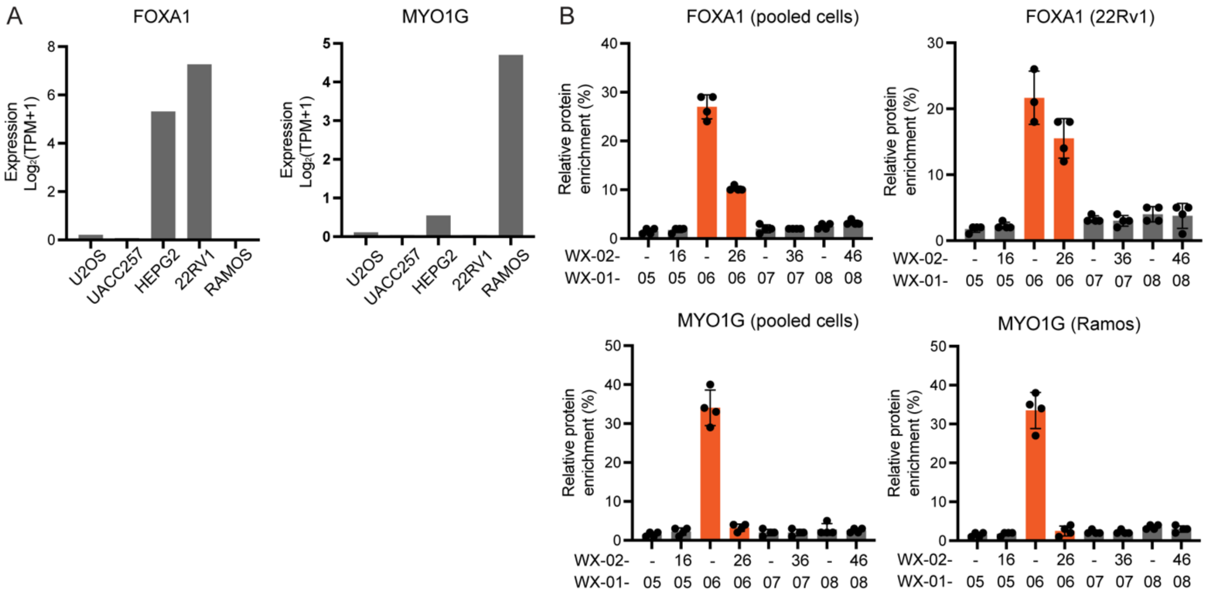

Figure S3. Quantification of proteins with cell line-restricted expression in protein-directed ABPP experiments performed with pooled cell lines. (A) Gene expression levels for two cell line-restricted proteins FOXA1 (left) and MYO1G (right) (RNA-seq data extracted from DepMap, 2023Q3 release).[7] (B) Protein-directed ABPP data for pooled cells vs individual cell lines[1] showing similar stereoprobe liganding profiles for FOXA1 and MYO1G. For B, data represent average values from four independent experiments; error bars represent s.d. values.

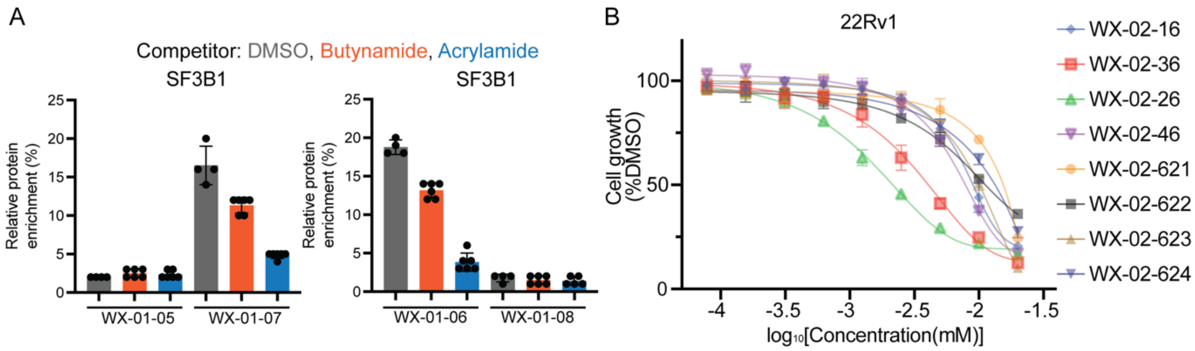

Figure S4. Tryptoline butynamides show attenuated engagement of the spliceosome factor SF3B1 and negligible stereoselective anti-proliferative effects. (A) Cross-competition protein-directed ABPP data for SF3B1, where enrichment was performed with alkyne tryptoline acrylamides. Pooled cell lines were treated with DMSO or parent tryptoline butynamides or acrylamides (20 µM) for 2 h followed by the stereomatched alkyne tryptoline acrylamide (5 µM) for 1 h. Workflow as shown in **Figure 2B**. (B) Concentration-dependent effects of tryptoline acrylamides and butynamides on the proliferation of 22Rv1 cells. Cells were treated with the indicated concentrations of compounds or DMSO, and, after 3 d, cell proliferation was determined by CellTiter-Glo (CTG). For A, data represent average values from four independent experiments. For B, data represent average values from three technical replicates from one experiment and are normalized to DMSO-treated cells. For A and B, error bars represent s.d. values.

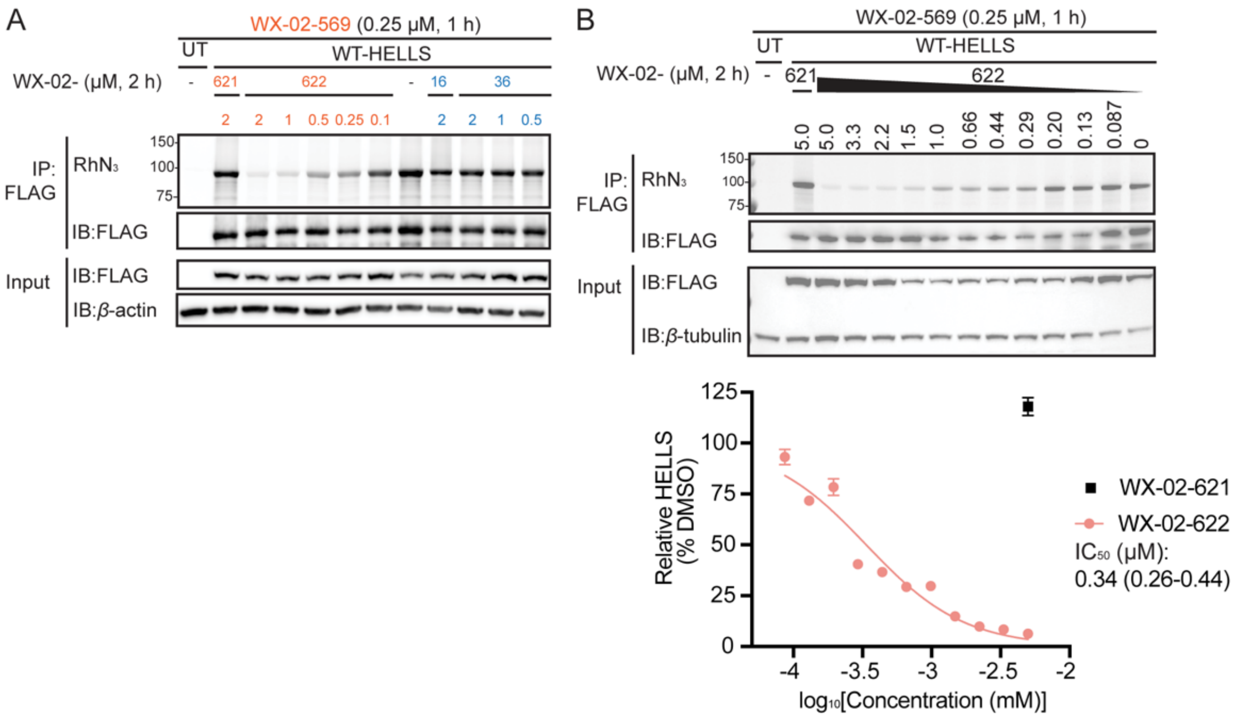

Figure S5. Further characterization of the butynamide-preferring protein HELLS. (A) Gel-ABPP data following anti-FLAG immunoprecipitation (IP) showing the stereoselective engagement of FLAG-tagged WT-HELLS by WX-02-569 (0.25 µM, 1 h), and the concentration-dependent blockade of WX-02-569-WT-HELLS interactions by pretreatment with tryptoline butynamide WX-02-622, but not with tryptoline acrylamide WX-02-36 (2 h). (B) Top, Gel-ABPP data following anti-FLAG IP showing the stereoselective engagement of FLAG-tagged WT-HELLS by WX-02-569 (0.25 µM, 1 h), and the concentration-dependent blockade of WX-02-569-WT-HELLS interactions by pretreatment with tryptoline butynamide WX-02-622 (2 h, 12-point concentration curve). Gel image is representative of two independent experiments. Bottom, quantification of concentration-dependent blockade of WX-02-569-WT-HELLS interactions by WX-02-622, from which an IC_{50} value of 0.34 µM (95% confidence interval of 0.26 to 0.44 µM) was calculated. Data are shown as average values ± s.d. from two independent experiments. IB: anti-FLAG, anti-*β*-actin, and anti-*β*-tubulin immunoblot; UT, untransfected cells.

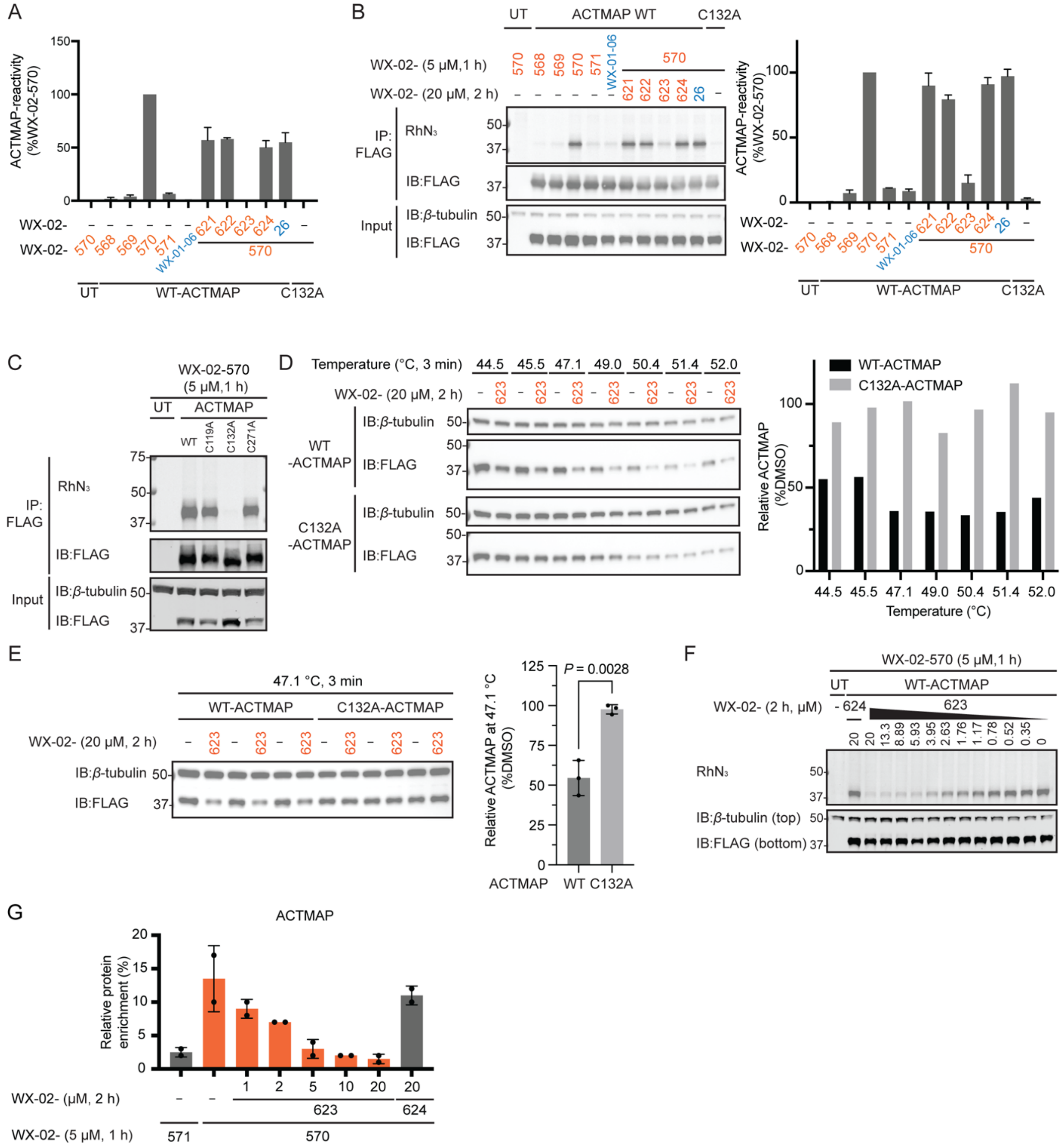

Figure S6. Further characterization of the butynamide-preferring protein C19orf54/ACTMAP. (A) Quantification of gel-ABPP data following anti-FLAG immunoprecipitation (IP) showing the stereoselective engagement of FLAG-tagged WT-ACTMAP, but not a C132A-ACTMAP mutant, by WX-02-570 (5 µM, 1 h), and the blockade of WX-02-570-WT-ACTMAP interactions by pretreatment with WX-02-623 (20 µM, 2 h) (see representative gel-ABPP data in **Figure 4C**). (B) Gel-ABPP data following anti-FLAG immunoprecipitation (IP) showing the stereoselective engagement of WT-ACTMAP, but not a C132A-ACTMAP mutant, by WX-02-570 (5 µM, 1 h) from *in vitro* (cell lysate) alkyne stereoprobe treatments and the blockade of this engagement by *in vitro* pretreatment with WX-02-623, but not other stereoprobes (20 µM, 2 h). (C) Gel-ABPP showing the engagement of WT-ACTMAP and C119A- and C132A-ACTMAP mutants, but not a

C132A-ACTMAP mutant, by WX-02-570 (5 µM, 1 h). (D) Western blot analysis from a thermal shift assay melt curve showing the temperature-dependent reduction in the solubility of WT-ACTMAP, but not C132A-ACTMAP following treatment with WX-02-623 (20 µM, 2 h) relative to DMSO control. (E) Replication of thermal stability effects of WX-02-623 at 47.1 °C for WT-ACTMAP, but not C132A-ACTMAP (P = 0.0028). Gels were loaded with the soluble fraction of HEK293T cells stably expressing ACTMAP proteins first treated with WX-02-623 (20 µM, 2 h) or DMSO in *vitro* at room temperature and then incubated at 47.1 °C for 3 min. (F) Gel-ABPP data showing concentration-dependent blockade of WX-02-570-WT-ACTMAP interactions by WX-02-623; image is representative of two independent experiments used to calculate and IC_{50} value for WX-02-623 (see **Figure 4E**). (G) Concentration-dependent engagement of endogenous ACTMAP by WX-02-623 measured by protein-directed ABPP. 22Rv1 cells were treated with the indicated concentrations of WX-02-623 or WX-02-624 for 2 h followed by WX-02-570 and WX-02-571, respectively (5 µM, 1 h) and analysis by protein-directed ABPP. IP, anti-FLAG immunoprecipitation; IB, anti-FLAG and anti-β-tubulin immunoblot; UT, untransfected cells. In A and B, data are average values ± s.d. from two independent experiments. In B, C and F, gel images are representative of two independent experiments. In D and E, gel images are from a single CETSA experiment. In E, data represent average values ± s.d. of three replicates from one experiment. In E, statistical significance was calculated with unpaired two-tailed Student's t-tests comparing WX-ACTMAP and C132A-ACTMAP band intensities normalized to DSMO in triplicates. In G, data are average values ± s.d. from two independent experiments.

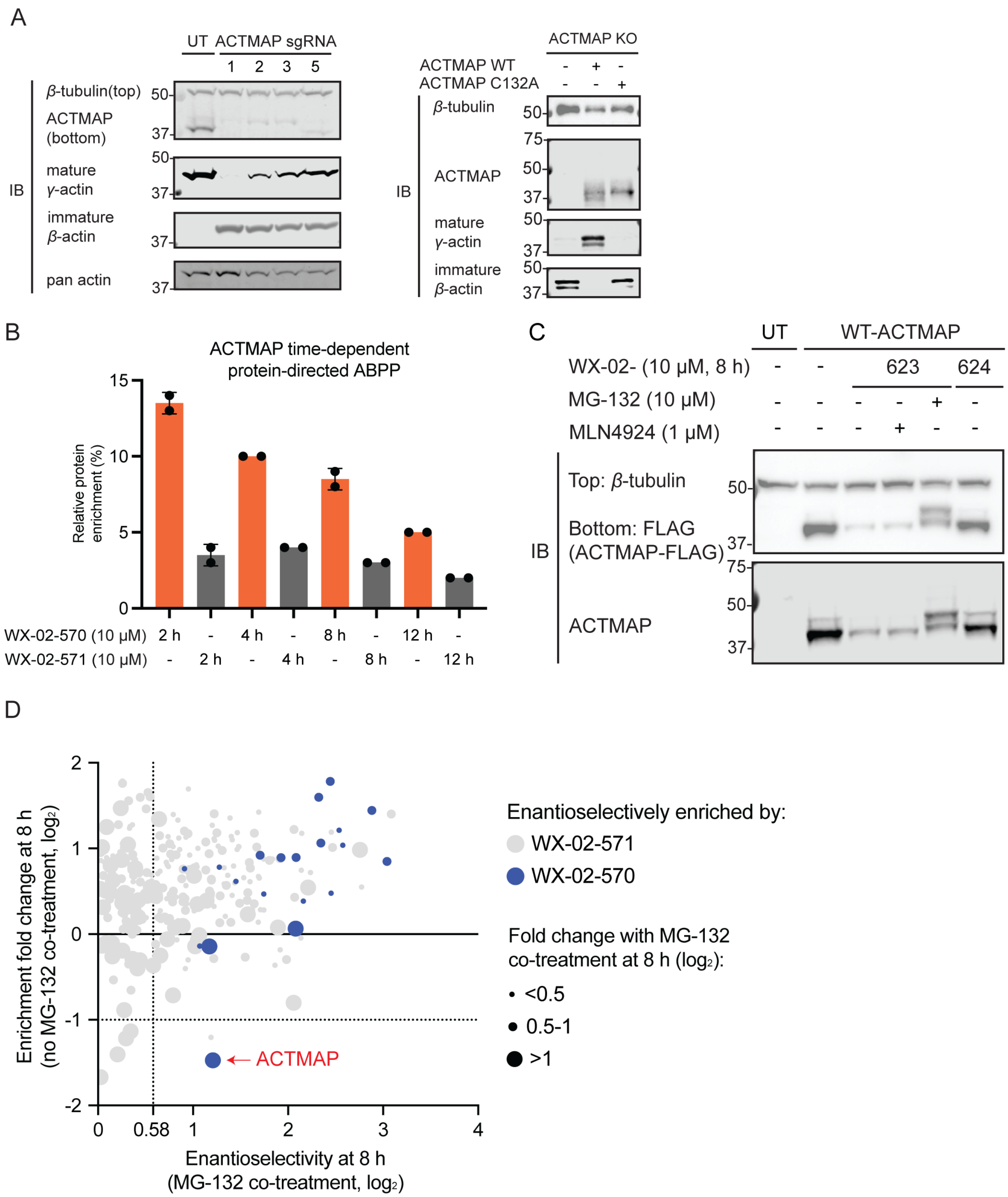

Figure S7. Characterization of the effects of tryptoline butynamide WX-02-623 on ACTMAP function in cancer cells. (A) Left, Western blots showing that the CRISPR/Cas9-mediated knockout (KO) of ACTMAP leads to accumulation of immature actin. Right, Western blots showing that, under ACTMAP-KO conditions, reconstitution of WT- ACTMAP, but not a C132A mutant, leads to depletion of immature actin. Due to lack of mature *β*-actin specific antibody, an antibody for mature *γ*-actin was used to measure actin maturation as previously reported.[8] (B) Protein-directed ABPP data showing evidence for the time-dependent degradation of ACTMAP.

22Rv1 cells were treated with alkyne tryptoline butynamide WX-02-570 or WX-02-571 (10 μM) for the indicated times. (C) Western blots showing ACTMAP degradation is rescued by co-treatment with MG-132, but not MLN4924. HEK293T cells recombinantly expressing FLAG epitope-tagged ACTMAP were co-treated with WX-02-623 (10 μM) and MLN4924 (1 μM) or MG-132 (10 μM) for 8 h. (D) Scatter plot showing the enantioselective enrichment values of proteins at 8 h with MG-132 co-treatment (*x*-axis) versus the fold-change in enrichment for these proteins with the preferred stereoprobe at 8 vs 2 h treatment without MG-132 co-treatment (*y*-axis). Dot size represents the fold change in enrichment by the indicated stereoprobe at 8 h with vs without MG-132 co-treatment. Proteins included in this plot were enantioselectively enriched (>3-fold) by either WX-02-570 (blue) or 571 (gray) at 2 h without MG-132 co-treatment. 22Rv1 cells were treated with 10 μM WX-02-570 or WX-02-571 for 2 or 8 h, with or without 10 μM MG-132 co-treatment. IB, anti-FLAG, anti-immature-*β*-actin, anti-*γ*-actin, anti-*β*-tubulin, anti-pan-actin, and anti-ACTMAP immunoblot; UT, untransfected cells. In A and C, gel images are representative of two independent experiments. In B, data are average values ± s.d. from two independent experiments. In D, data are average values from two independent channels in one MS experiment.

Reagents and Materials

Table S2: Reagents and materials used in this study.

Reagents/materials	Source	Catalog number
DMEM (Dulbecco's Modified Eagle's Medium)	Corning	15-013-CV
RPMI 1640 1×	Corning	15-040-CV
Desthiobiotin polyethyleneoxide iodoacetamide (IA-DTB)	Santa Cruz Biotechnology	sc-300424
Biotin-PEG4-azide	BroadPharm	BP-22119
Urea	Fisher Scientific	M1084871000
Iodoacetamide	Sigma-Aldrich	I1149
Dithiothreitol (DTT)	Fisher Bioreagents	BP172
Tris(2-carboxyethyl)phosphine HCl (TCEP)	Biosynth	FT01756
Sequencing Grade Modified Trypsin	Promega	V5111
Streptavidin Agarose Resin	Fisher Scientific	20353
High Capacity Streptavidin Agarose	Fisher Scientific	20361
Micro Bio-Spin column	Bio-Rad	7326204
NonidetTM P40 substitute (IgepalTM CA-630)	USB Corporation	19628
Triethylammonium bicarbonate buffer pH 8.5	Sigma-Aldrich	T7408
TMT10plex™ Isobaric Label Reagent Set	ThermoFisher Scientific	90406
TMTpro™ 16plex Label Reagent Set	ThermoFisher Scientific	A44520
Ammonium bicarbonate	Sigma-Aldrich	09830
Hydroxylamine solution 50 wt. % in water	Sigma-Aldrich	467804
Formic acid, ~98%, for mass spectrometry	Honeywell Fluka	94318-250ML-F
Methanol LC-MS Grade	Fisher Scientific	A452SK-4
Acetonitrile Optima™LC/MS Grade	Fisher Scientific	A955
Water Optima™ LC/MS Grade	Fisher Scientific	W64
Protein LoBind Tubes 1.5 mL	Fisher Scientific	13-698-794
Protein LoBind Tubes 2.0 mL	Fisher Scientific	13-698-795
Clear 384 Well Polystyrene Plates With F-Bottom	Greiner Bio	781101
DMSO	Corning	25-950-CQC
Puromycin	Invivogen	ant-pr-1
Blasticidin	Invivogen	ant-bl-1
EPPS	Millipore	E0276
Sodium Dodecyl Sulfate (SDS) Biotechnology grade, 1kg	BioPioneer	C0065
Glycerol	Fisher Scientific	BP229-4
Chloroform	Fisher Scientific	C297-4
Tris(benzyltriazolylmethyl)amine (TBTA)	TCI	T2993
Fetal Bovine Serum, Dialyzed	Omega Scientific	FB-03

One Shot Stbl3 Chemically Competent E. coli	Life Technologies	C737303
cOmplete ULTRA Tablets, Protease Inhibitor Cocktail	Roche	5892970001
NuPAGE LDS Sample Buffer (4×)	Invivogen	NP0008
2-Mercaptoethanol, 99%	Acros Organics	125472500
SuperSignal™ West Pico PLUS Chemiluminescent Substrate	ThermoFisher Scientific	34580
Q5® Site-Directed Mutagenesis Kit	New England Biolabs	E0552S
Pierce™ Anti-DYKDDDDK Magnetic Agarose	ThermoFisher Scientific	A36797
FuGENE® HD Transfection Reagent	Promega	E2311
Lenti-X™ Concentrator	Takara Bio	631231
Triton™ X-100	Sigma-Aldrich	T8787
PEI MAX® - Linear Polyethylenimine Hydrochloride (MW 40,000)	Polysciences	24765
Gateway™ LR Clonase™ II Enzyme mix	Invitrogen	11791020
BsmBI-v2	New England Biolabs	R0739S
InstantBlue® Coomassie Protein Stain (ISB1L)	abcam	ab119211
Power Blotter Select Transfer Stack, nitrocellulose, mini	ThermoFisher Scientific	PB3210
Power Blotter Select Transfer Stack, nitrocellulose, regular	ThermoFisher Scientific	PB3310
Power Blotter Select Transfer Stack, PVDF, mini	ThermoFisher Scientific	PB5210
Power Blotter Select Transfer Stack, PVDF, regular	ThermoFisher Scientific	PB5310
GlutaMAX™ Supplement	Fisher Scientific	35050061
Pierce™ BCA Protein Assay Kits	ThermoFisher Scientific	23225
Trypsin-EDTA (0.25%), phenol red	Gibco™	25200114
Rabbit Anti-immature beta actin antibody	Gift from Dr. Thijn Brummelkamp	PMID: 36173861
Mouse Monoclonal Anti-GAPDH HRP-conjugated antibody	Proteintech	HRP-60004
Mouse Monoclonal Anti-FLAG(R) M2-HRP antibody	Sigma-Aldrich	A8592
Mouse Monoclonal Anti-gamma Actin antibody (2A3)	abcam	ab123034
Rabbit Polyclonal Anti-C19orf54/ACTMAP antibody	Sigma-Aldrich	HPA059043
Rabbit Monoclonal Anti-pan-actin antibody (D18C11)	Cell Signaling	#8456
Rabbit Monoclonal Anti-beta tubulin (2O13) recombinant HRP-conjugated antibody	Proteintech	HRP-80713
Mouse Monoclonal Anti-beta actin antibody (C4) HRP-conjugated antibody	Santa Cruz	sc-47778 HRP
Donkey IRDye® 800CW anti-rabbit IgG secondary antibody	LI-COR Biotech	926-32213
Goat IRDye 680RD anti-mouse IgG secondary antibody	LI-COR Biotech	926-68070
MG-132	Selleck Chemicals	S2619
MLN4924	Cell Signaling	#85923

Experimental Methods

Cell culture

Cell lines used in this study were acquired from the American Type Culture Collection (ATCC), National Cancer Institute Developmental Therapeutics Program (NCI-DTP), or Takara and cultured in a humidified incubator at 37 °C with 5% CO_2. Ramos (ATCC, CRL-1596), 22Rv1 (ATCC, CRL-2505), UACC-257 (NCI-DTP, CVCL_1779), U-2 OS (ATCC, HTB-96), and Hep G2 (ATCC, HB-8065) cell lines were maintained in RPMI-1640 supplemented with 10% FBS, 2 mM GlutaMAX™, 100 U/mL penicillin, and 100 µg/mL streptomycin. HEK293T (ATCC, CRL-3216) and Lenti-X (Takara, 632180) cells were maintained in DMEM supplemented with 10% FBS, 2 mM GlutaMAX™, 100 U/mL penicillin, and 100 µg/mL streptomycin. All cell lines used for pooled ABPP studies were parental in origin with the exception of UACC-257 cells, which stably expressed a C80S-TOE1 mutant protein for use in unrelated studies.[9]

Gel-ABPP for assessing proteome-wide reactivity of stereoprobes

Ramos cells (1 mL, 3 million cells/mL) were treated with alkyne stereoprobes for 1 h at 37 ˚C. Cells were harvested by centrifugation at 800 × *g* and then washed 2 × 1 mL with ice-cold Dulbecco's phosphate-buffered saline (DPBS). The pellets were resuspended in 200 µL ice-cold DPBS and lysed by probe sonication (2 × 15 pulses, 10% power output). Samples were normalized to 1 mg/mL in 50 µL with the Pierce™ BCA Protein Assay Kit relative to a bovine serum albumin (BSA) standard according to the manufacturer's protocol. Click reactions[2, 3] were subsequently performed by adding 5 µL 'click' mixture [3 µL of 1.7 mM tris[(1-benzyl-1H-1, 3-triazol-4-yl)methyl]amine (TBTA) in 4/1 *t*-BuOH/DMSO v/v, 1 µL of 50 mM $CuSO_4$ in HPLC H_2O, 1 µL of 1.25 mM rhodamine-PEG$_3$-azide in DMSO, 1 µL of freshly prepared 50 mM tris-(2-carboxyethyl)phosphine hydrochloride (TCEP) in HPLC water] for 1 h followed by the addition of 14 µL 4× LDS gel loading buffer containing 5% v/v 2-mercaptoethanol (BME). Samples were denatured at 95 ˚C, resolved at 275 V for 240 min on a 10% tris-glycine gel, and the in-gel fluorescence was measured with a ChemiDoc MP imaging system using the rhodamine filter set. Gels were then stained with Coomassie Instant Blue™ for 10 min at room temperature, destained for 5 min in water, and visualized with a ChemiDoc MP imaging system using the Coomassie filter. Images were processed using Image Lab software (version 6.1.0).

Protein-directed ABPP in pooled cells

***In situ* compound treatment and sample processing.** Ramos, 22Rv1, UACC-257, U-2 OS, and Hep G2 cells were seeded and cultured separately under standard conditions. On the day of treatment, adherent cell lines were detached with trypsin-EDTA (0.25%) at 37 ˚C for 5 min and quenched with complete media before pelleting at 800 × *g* for 5 min and resuspending in complete media. Suspension cell lines were pelleted at 800 × *g* for 5 min and resuspended in complete media. Cell lines were then counted on a TC20 automated cell counter with trypan blue (1:1 v/v) and pooled in the appropriate ratio to yield equal protein content per cell line, as measured by Pierce™ BCA Protein Assay Kit relative to a BSA standard. For 1 mg protein per channel: Ramos: 3.75 million/channel; 22Rv1: 1.55 million cells/channel; Hep G2: 0.9 million/channel; UACC-257: 0.54 million/channel; U-2 OS: 1.05 million/channel. Pooled cells were treated as a 10 mL suspension with DMSO or 20 µM competitor stereoprobes (0.1% DMSO v/v final) for 2 h with mixing every 15 min. The cells were then treated with 5 µM stereochemically matched alkyne stereoprobes (0.1% DMSO v/v final) for 1 h with mixing every 15 min. The cells were washed 3 × 10 mL with ice-cold DPBS by centrifuging at 800 × *g* for 5 min, transferred into 1.5 mL tubes, and stored at −80 °C. Protein-directed ABPP (i.e., cell lysis, normalization, copper-catalyzed 'click' chemistry-based ligation to biotin-PEG$_4$-azide, streptavidin enrichment, TMTpro16 tag-based multiplexing, Sep-Pak desalting, preparatory HPLC-based high-pH fractionization and LC-MS analysis) was carried out as previously reported[1, 10, 11] with the following minor modifications. Pierce™ High Capacity Streptavidin Agarose beads were used to capture liganded proteins for 2 h at room temperature with rotation and, after washing, enriched proteins were digested for 16 h with 75 µL of trypsin mix (200 mM EPPS pH 8.0 supplemented with 1 M urea, 1 mM $CaCl_2$ and 20 µg/mL trypsin) at 37 ˚C with shaking. The beads were pelleted at 5,000 × *g* for 2 min at

room temperature, the supernatant was collected, diluted with 25 µL of acetonitrile (25% v/v final), and labeled with 5 µL of a 20 mg/mL solution of the corresponding TMTpro16 plex tag in anhydrous acetonitrile for 1 h at room temperature with vortexing every 30 min.

TMT liquid chromatography-mass-spectrometry (LC-MS) analysis. All LC-MS analysis and data analysis were performed as described previously.[1, 6, 10, 11] Briefly, samples were run on an Orbitrap Eclipse Tribrid Mass Spectrometer (Thermo Scientific) or an Orbitrap Fusion Tribrid Mass Spectrometer (Thermo Scientific) coupled to an UltiMate 3000 Series Rapid Separation LC system and autosampler (Thermo Scientific Dionex).

Data processing. Raw files were uploaded to the Integrated Proteomics Pipeline (IP2, version 6.0.2) available at http://ip2.scripps.edu/ip2/mainMenu.html, and MS2 and MS3 files were extracted from the raw files using RAW Converter (version 1.1.0.22, available at http://fields.scripps.edu/rawconv/) and searched using the ProLuCID algorithm using a reverse concatenated, non-redundant variant of the Human UniProt database (release 2016-07). Cysteine residues were searched with a static modification for carboxyamidomethylation (+57.02146 Da) and methionine residues were searched with a differential modification for oxidation (+15.9949 Da). Enrichment ratios (probe versus probe) were calculated for each peptide–spectra match by dividing each TMT reporter ion intensity by the sum intensity for all the channels. Peptide–spectra matches were then grouped based on protein ID and (excluding peptides with summed reporter ion intensities less than 10,000, coefficient of variation of greater than 0.5, and less than 2 distinct peptides). Replicate channels were grouped across each experiment and average values were computed for each protein. A variability metric was also computed across replicate channels, which equaled the ratio of the standard deviation to the mean for channels corresponding to the highest enriching probe and was expressed as a percentage. A protein was considered stereoselectively enriched if it was (1) quantified in two independent experiments, (2) the average enrichment by the alkyne stereoprobe was greater than 3-fold that of its enantiomer and (3) if the variability corresponding to the alkyne stereoprobe with the highest enrichment was less than or equal to 30%. A protein was considered stereoselectively liganded if enrichment was greater than 2-fold the enrichment observed following treatment with a stereochemically matched, non-alkyne competitor stereoprobe (i.e. 50% blockade of enrichment in competitor-treated channels as compared to DMSO-pretreated channels).

Cross-competition ABPP in pooled cells

***In situ* compound treatment and sample processing.** Pooled, suspended cells were prepared to the “Protein-directed ABPP in pooled cells” protocol and then treated with DMSO, 20 µM non-alkyne acrylamide stereoprobes, or 20 µM non-alkyne butynamide stereoprobes (0.1% DMSO v/v) for 2 h at 37°C with mixing every 15 min. The cells were then treated with 5 µM stereochemically matched alkyne stereoprobes (acrylamide or butynamide) for 1 h at 37°C with mixing every 15 min. Subsequent collection and sample processing were performed as described in “Protein-directed ABPP in pooled cells.”

Data processing. Enrichment ratios and competitions were calculated using the same workflow as in “Protein-directed ABPP in pooled cells.” A protein was considered butynamide-preferring if it showed greater than 2-fold blockade of enrichment by the parent butynamide competitor that was also 1.5-fold greater blockade than that of the stereo-matched parent acrylamide competitor; whereas a protein was considered acrylamide-preferring it showed greater than 2-fold blockade of enrichment by the parent acrylamide competitor that was also 1.5-fold greater blockade than that of the stereo-matched parent butynamide competitor. The shared targets show greater than 2-fold blockade of enrichment by both the non-alkyne butynamide and acrylamide competitors. Inconclusive targets are those with less than 50% blockade by either non-alkyne acrylamide or butynamide in cross-competition ABPP or showing conflicting blockade values when enriched by acrylamide or butynamide alkynes (e.g., acrylamide-preferring when enriched with acrylamide alkyne while butynamide-preferring when enriched with butynamide alkyne).

Cysteine-directed ABPP in pooled cells

***In situ* treatment and sample processing.** Pooled, suspended cells were prepared to the "Protein-directed ABPP in pooled cells" protocol and then treated with DMSO or 20 μM tryptoline butynamide stereoprobes for 3 h at 37 °C in suspension state with mixing every 15 min. Subsequent sample collection was performed as described in "Protein-directed ABPP in pooled cells." Cysteine-directed ABPP was carried out as previously reported.[1, 6, 10, 11]

Data processing. Raw files were uploaded to the Integrated Proteomics Pipeline (IP2, version 6.0.2) available at http://ip2.scripps.edu/ip2/mainMenu.html, and MS2 and MS3 files were extracted from the raw files using RAW Converter (version 1.1.0.22, available at http://fields.scripps.edu/rawconv/) and searched using the ProLuCID algorithm using a reverse concatenated, non-redundant variant of the Human UniProt database (release 2016-07). Cysteine residues were searched with a static modification for carboxyamidomethylation (+57.02146 Da). A dynamic modification for IA-DTB labeling (+398.25292 Da) was included with a maximum number of two differential modifications per peptide. Cysteine engagement ratios (DMSO versus compound) were calculated for each peptide–spectra match by dividing each TMT reporter ion intensity by the average intensity for the DMSO channels. Peptide–spectra matches were then grouped based on protein ID and residue number (for example, AGPS_C190), excluding peptides with summed reporter ion intensities for the DMSO channels of less than 10,000 and a coefficient of variation for DMSO channels of greater than 0.5. Replicate channels were grouped across each experiment, and average values were computed for each cysteine. A variability metric was also computed across replicate channels, which equaled the ratio of the median absolute deviation to the average and was expressed as a percentage. A cysteine site was considered enantioselectively liganded if the variability corresponding to the probe leading to the highest blockade of IA-DTB was less than or equal to 20%, and at least one of the following additional criteria were met: (1) the average IA-DTB blockade by the probe was greater than 50% and greater than 1.5-fold of its enantiomer, and protein was stereoselectively liganded by protein-directed ABPP, or (2) IA-DTB blockade by the probe was greater than 66.7% and greater than 2.5-fold that of its enantiomer, and the same probe led to less than 25% IA-DTB blockade of at least one other cysteine in the same protein. Tryptoline acrylamide data were from previously published data and processed in the same manner.

Targeted Lys-C digested ABPP

***In vitro* treatment and sample processing.** HNT-34 (DSMZ, ACC 600) cells were lysed and normalized to 2.5 mg/mL. 500 μg total protein was titrated with varying concentrations of WX-02-621 or WX-02-622 (500–0.8 μM, 5-fold dilutions in duplicates) for 1 h at room temperature. Compound treated lysates were then incubated with DBIA probe for 1 h at room temperature. Samples were digested with LysC and processed with standard protocol described in "Cysteine-directed ABPP." Targeted data acquisition of the HELLS_364 peptide was performed via parallel reaction monitoring (PRM) on an Exploris Orbitrap 120, as previously described.[12, 13] Briefly, precursor ions corresponding to an IA-DTB-modified HELLS_364 peptide (amino acids 364–371, +3 charge state, m/z 452.28) were isolated and fragmented by high energy collision-induced dissociation and fragments were detected in the Orbitrap at 17,500 resolution. The resulting PRM data were analyzed using Skyline and quantification was performed by summing the peak areas corresponding to six fragment ions from each peptide.

Data processing. Raw AUCs were normalized using RTS peptides (#2–11) and target engagement was calculated relative to DMSO controls (n = 16).

IP-MS

***In situ* treatment and sample processing.** Cells stably expressing FLAG epitope-tagged protein of interest (HEK293T ACTMAP WT or C132A) were seeded at 5 million cells per 10 cm plate the day prior to treatment. The media was removed and replaced with fresh media containing DMSO or 10 μM stereoprobes (0.1% DMSO v/v) for 3 h. The cells were detached with trypsin-EDTA (0.25%), quenched with complete DMEM media, collected by centrifugation at 500 × g for 5 min, and washed 3 × 10 mL with ice-cold DPBS. Cell pellets were stored at −80 ˚C, thawed in 1 mL of IP lysis buffer [50 mM EPPS pH 8.0 supplemented

with 150 mM NaCl, 1% Triton™ X-100 v/v, 10% glycerol v/v, and one EDTA-free cOmplete protease inhibitor per 10 mL], and lysed by rotating for 1 h at 4 °C. The lysate was clarified by centrifugation at 16,000 × *g* for 5 min at 4 ˚C and the supernatant was normalized to 1–3 mg in 1 mL with the Pierce™ BCA Protein Assay Kit relative to a BSA standard. Each sample was treated with 40 µL prewashed anti-FLAG magnetic beads for 3 h at 4 ˚C with rotation. The samples were washed 3 × 1 mL with IP wash buffer (25 mM EPPS pH 8.0 supplemented with 150 mM NaCl, 0.2% Triton™ X-100 v/v) and 1 × 1 mL with 50 mM EPPS pH 8.0 using a DynaMag-2 magnetic rack. Enriched proteins were eluted in 40 µL 50 mM EPPS pH 8.0 supplemented with 8 M urea at 65 ˚C for 10 min. The supernatant was collected and reduced by adding 2 µL 200 mM DTT in HPLC water for 15 min at 65 ˚C and alkylated by adding 2 µL 400 mM iodoacetamide in HPLC water for 30 min at 37 ˚C. Samples were diluted to 2 M urea by adding 115 µL 50 mM EPPS pH 8.0 and digested with 4 µL trypsin mix (50 mM EPPS supplemented with 25 mM $CaCl_2$ and 0.25 µg/µL trypsin) overnight at 37 ˚C with shaking. Acetonitrile (75 µL) was added, and the samples were multiplexed with TMTpro 16plex and desalted as described in "Protein-directed ABPP in pooled cells." The sample was then high-pH spin column fractionated and pooled into three fractions as previously described.[1, 10, 11]
Data processing. IP-MS data were processed in the same manner as described in "Protein-directed ABPP pooled cells" except protein signals were normalized to the FLAG epitope-tagged protein pulled down within each treatment group and then to DMSO signals across treatment groups, unless otherwise noted.

Time-course and concentration-dependent protein-directed ABPP
10 million 22Rv1 cells were seeded per 10 cm plate 12 h before treatment. Cells were treated with alkynes at 10 µM for 2–12 h (time-course) or 1–20 µM (concentration screen) for 2 h before adding 5 µM alkyne for 1 h. The samples were collected and processed in the same workflow as described in "Protein-directed ABPP in pooled cells." For experiments performed with MG-132, 10 million 22Rv1 cells were seeded per 10 cm plate 12 h before treatment. Cells were treated with alkynes at 10 µM for 2 or 8 h, with or without 10 µM MG-132 co-treatment. The samples were collected and processed in the same workflow as described in "Protein-directed ABPP in pooled cells."
Data processing. Processing and analysis of MS data followed the "Protein-directed ABPP in pooled cells." For time-course protein-directed ABPP experiments, only proteins that were enantioselectively enriched by WX-02-570 or WX-02-571 in "Protein-directed ABPP in pooled cells" were analyzed and plotted for enrichment changes at 8 h versus 2 h. For time-course protein-directed ABPP experiments, with MG-132 co-treatment, only proteins that were enantioselectively enriched by WX-02-570 or WX-02-571 in the 2 h channels without MG-132 co-treatments were analyzed and plotted.

Cloning and mutagenesis
AGPS plasmids were obtained from GenScript in pcDNA3.1-C-(k) DYK (FLAG). C19orf54/ACTMAP and HELLS plasmids were obtained from Twist Biosciences after codon optimization with C-FLAG in entry clones (pTwist ENTR). Mutagenesis was performed with Q5® Site-Directed Mutagenesis Kit, using primers shown below. Wild-type and cysteine-to-alanine/serine mutants of C-terminally FLAG-tagged HELLS and ACTMAP were prepared by Gateway cloning from pTwist ENTR into lentiviral vector pLX304 (Addgene, 25890) using Gateway™ LR Clonase™ II Enzyme mix. sgRNAs of ACTMAP (sequences reported below) were annealed and cloned into LentiCRISPR-v2 (Addgene, 52961) using a previously reported protocol[14] except BsmBI was from NEB and the digestion was performed according to the manufacturer's protocol.

Table S3: Primers used for Q5® Site-Directed Mutagenesis.

Gene	Mutant	Forward primer (5'– 3')	Reverse primer (5'– 3')
HELLS	C364S	GAACATGAAGAGCCGTCTCATCC	TTAATGCGATGTCCC
ACTMAP	C132A	GGGCCCTCAAGCGGGTCTGGTGG	TCTTGAATCAAACTCGGAAG

ACTMAP	C119A	GATTCTGTTTGCGGCCGACCTTCCG	CACCTCAGGTCTCCTC
ACTMAP	C271A	TGGTACACCCGCGCAACCACCAAG	AGCACTGGGTGGAAG
AGPS	C190A	TCATGGTCATGCGCTTCATGAGATATTTTTGC	GCTCTAAATACTCGATCATC

Table S4: sgRNAs cloned into LentiCRISPR-v2 for ACTMAP knockout.

ACTMAP	sgRNA
sgRNA1	ACCCGTATGAGTCTCTCCAG
sgRNA2	GGACGCCACTGGGGGGCGAC
sgRNA3	GAAACTACACAAGGACCGGT
sgRNA5	ACCGGGCCGGCCTTATCAA

Generation of ACTMAP stable HEK293T cell lines

Lentivirus was prepared by seeding 4 million Lenti-X 293T cells in 10 cm dishes in 8 mL media the day before transfection. 3 µL of FuGENE HD was dissolved in 160 µL OPTI-MEM medium followed by protein-encoding viral vector (3.4 µg), lentiviral packaging vector (pCMV-dR8.91, 1.7 µg) and envelope vector (VSV-G, 0.85 µg). The transfection mix was incubated at room temperature for 10 min before adding dropwise to the Lenti-X 293T cells. The media containing virus was collected 72 h post-transfection, filtered through a 0.45-µm syringe filter, diluted 3:1 with Lenti-X Concentrator and incubated at 4 ˚C with rotation for 2 h. The virus was concentrated by centrifugation at 1,500 × *g* at 4 ˚C for 45 min. The viral pellet was resuspended in 10% volume of original volume with fresh DMEM media. For each transduction, 0.5–1 million HEK293T cells were mixed with 100–200 µL of concentrated viral supernatant in 1–2 mL DMEM media supplemented with 8 µg/mL polybrene in a 12-well plate. HEK293T cells were spin-infected at 1,000 × *g* at 30 ˚C for 1–2 h and incubated for 24 h at 37 ˚C. Selection was initiated with 8 µg/mL blasticidin for 1–2 weeks until a negative control had no surviving cells. Successful transductions were confirmed by FLAG immunoblots (Western blotting method is described in following section "Gel-ABPP with recombinant proteins without immunoprecipitation") and reselected with 8 µg/mL blasticidin if thawing from frozen stocks.

Generation of ACTMAP CRISPR/Cas9 knockout cells

Viral supernatants were generated by the transduction of cells with LentiCRISPR-v2 carrying sgACTMAP and stable knock-out cells were generated as described in "Generation of ACTMAP stable cell lines." Stably knocked-out ACTMAP cell lines were selected with 1 µg/mL of puromycin for one week until a negative control had no surviving cells. KO populations were confirmed by immunoblot with anti-ACTMAP antibody and reselected with 1 µg/mL of puromycin if thawing from frozen stocks.

Gel-ABPP with recombinant proteins without immunoprecipitation

0.5 million HEK293T cells were seeded per 6-well dish the day before transfection. FLAG-epitope tag plasmids (1–2 µg) were incubated at room temperature with polyethylenimine (PEI, 40,000 Da), 3:1 ratio to plasmid) in 200 µL OPTI-MEM medium for 15 min. The mixture was added dropwise to the cells and allowed to incubate for 24 h under standard culture conditions. Cells were treated with DMSO or with competitor probes for 2 h, followed by alkyne probes for another 1 h. The cells were then detached by trypsin-EDTA (0.25%), quenched with complete DMEM media, collected at 500 × *g* for 5 min and washed 2 × 1 mL with ice-cold DPBS. Cell pellets were resuspended in 100 µL of ice-cold IP lysis buffer [50 mM EPPS pH 8.0 supplemented with 150 mM NaCl, 1% Triton™ X-100 v/v, 10% glycerol v/v, and one EDTA-free cOmplete protease inhibitor per 10 mL], lysed by sonication (1 × 15 pulses, 10% power output) and centrifuged at 16,000 × *g* for 5 min. Supernatants were normalized to 1 mg/mL in 50 µL using the Pierce™ BCA Protein Assay Kit relative to a BSA standard and treated with 5 µL of click mix as described in "Gel-

ABPP for proteome-wide reactivity.” The click reaction was quenched by the addition of 18 µL of 4× LDS gel loading buffer containing 5% BME and boiled at 95 ˚C for 5 min. The samples were resolved on a 12% tris-glycine SDS–PAGE gel at 160 V for 60 min and imaged by in-gel fluorescent scanning using a ChemiDoc MP imaging system using the rhodamine filter set. Proteins were transferred onto nitrocellulose or PVDF membrane using Power Blotter (1.3 A each gel for 6 min), blocked with 5% non-fat milk in TBST for 1 h at room temperature or overnight at 4 ˚C. Membranes were incubated with anti-FLAG antibody (HRP-conjugate, 1:2,000 in 5% milk in TBST) or the loading controls anti-GAPDH, anti-β-actin , or anti-β-tubulin (HRP-conjugates, 1:5,000 in 5% milk in TBST) for 1 h at room temperature or overnight at 4 ˚C. The membranes were washed three times with TBST for 5 min at room temperature and were developed with enhanced chemiluminescent western blot substrate and fluorescence was measured on a ChemiDoc MP imaging system using the chemiluminescence filter set. Images were processed using Image Lab software (version 6.1.0).

IP-based Gel-ABPP with recombinant proteins

HEK293T cells were seeded and transfected with C-terminally FLAG-tagged AGPS, HELLS, or ACTMAP mutants according to “Gel-ABPP with recombinant proteins without immunoprecipitation.” 1 million HEK293T cells stably expressing C-terminally FLAG-tagged ACTMAP were seeded per 6-well the day before treatment. The cells were treated and collected as described in “Gel-ABPP with recombinant proteins without immunoprecipitation.” Cell pellets were resuspended in 100–200 µL IP lysis buffer [50 mM EPPS pH 8.0 supplemented with 150 mM NaCl, 1% Triton™ X-100 v/v, 10% glycerol v.v, and one tablet EDTA-free cOmplete protease inhibitor per 10 mL], lysed by probe sonication (1 × 15 pulses, 10% power output) and centrifuged at 16,000 × *g* for 5 min. Protein concentrations were normalized in IP lysis buffer to 0.5–1 mg/mL in 200 µL with the Pierce™ BCA Protein Assay Kit relative to a BSA standard. 20 µL was removed for the input and treated with 2 µL click mix from “Gel-ABPP with recombinant proteins without immunoprecipitation”) for the 1 h before quenching with 5 µL of 4× LDS gel loading buffer containing 5% BME at 95 ˚C for 10 min. The remaining lysate (180 µL) was mixed with 10 µL of anti-FLAG magnetic beads (prewashed with 2 × 1 mL IP wash buffer and resuspended in the original volume in IP lysis buffer) and incubated at 4 ˚C with rotation for 1–2 h. The samples were washed 2 × 1 mL with ice-cold IP wash buffer (25 mM EPPS pH 8.0 supplemented with 150 mM NaCl, 0.2% Triton™ X-100 v/v) and 1 × 1 mL with ice-cold DPBS. Beads were resuspended in 20 µL DPBS for on-bead click reaction with 3 µL of click mix (same mix as “Gel-ABPP with recombinant proteins without immunoprecipitation”) for 1 h at room temperature with vortexing every 15 min. The click reactions were quenched with 5 µL of 4× LDS gel loading buffer containing 5% BME and the enriched proteins were eluted by boiling at 95 ˚C for 5 min. The supernatant was collected with a magnetic stand and the samples were resolved on 12% tris-glycine SDS–PAGE gels at 160 V for 60 min. Input and IP samples were imaged and blotted as described in “Gel-ABPP with recombinant proteins without immunoprecipitation.”

Cell proliferation assays

5,000 22Rv1 cells were seeded per 96-well in 50 µL RPMI media 24 h before treatment. 50 µL of media containing 2× stereoprobes or DMSO (0.078–20 µM final concentrations, 0.1% DMSO v/v) was added and allowed to continue incubating for 72 h. Cell proliferation was measured by 30 µL CellTiter-Glo (CTG) reagent on CLARIOstar Plate Reader (software version 5.40 R3, firmware version 1.21). Raw values were normalized to DMSO-treated wells and analyzed using GraphPad PRISM (software version 10.4.2).

Western Blots for ACTMAP functional assays

3 million 22Rv1 cells or 1 million HEK293T stably expressing WT-ACTMAP or C132A-ACTMAP were seeded per 6-well 12 h before treatment. After changing the media, cells were treated with WX-02-623/624 (1–20 µM 48 h for concentration-dependent WB, 10 µM 2–48 h for time-course WB, 10 µM 2–24 h for proteasome inhibitors co-treatment, final 0.1% DMSO v/v) with or without degradation inhibitors (1000× dilution, 2–24 h) and were collected and washed as described in “Gel-ABPP with recombinant proteins without immunoprecipitation.” Cell pellets were frozen at – 80 ˚C or directly processed as previously

described with minor modifications.[8] Briefly, cells were lysed in 100 µL DPBS, normalized to 2 mg/mL in 50 µL with the Pierce™ BCA Protein Analysis Kit relative to a BSA standard, and denatured by adding an equal volume 4× LDS sample buffer containing 200 mM DTT (2× LDS and 100 mM DTT final). The samples were heated at 95 ˚ C for 5 min before resolving on a 12% tris-glycine SDS-PAGE at 160 V for 60 min. The proteins were then transferred onto nitrocellulose membranes using Power Blotter (1.3 A each gel for 6 min). The membranes were blocked with 5% non-fat milk in TBST before incubating with primary antibody (anti-C19orf54/ACTMAP, 1:1,000; anti-γ-actin, anti-pan-actin, anti-β-tubulin, and anti-immature β-actin, 1:5,000, in 5% milk in TBST) for 2 h at room temperature or overnight at 4 ˚C. The membranes were washed three times with TBST for 5 min before incubating with the corresponding anti-mouse (680RD) or rabbit (800CW) secondary IRDye antibody (1:5,000 in 5% milk in TBST) for 1 h at room temperature. The membranes were washed three times with TBST for 5 min before imaging with an Odyssey CLx Imager (LI-COR Biotech) and images were processed using Image Studio 1.0.20.

Thermal shift assay

HEK293T stably expressing WT-ACTMAP or C132A-ACTMAP were lysed in DPBS with EDTA-free cOmplete protease inhibitor (one tablet per 10 mL). Cell lysates was normalized to 4 mg/mL with the Pierce™ BCA Protein Analysis Kit relative to a BSA standard and incubated with compounds (20 µM) or DMSO for 2 h at room temperature and then transferred into PCR tubes in 30 µL aliquots. The tubes were heated in a thermal cycler for 3 min from 44.5 to 52 ˚ C (for melt curve) or at 47.1 ˚C (for stability effects), cooled to room temperature for 3 min, and then placed on ice. The samples were diluted to a final volume of 60 µL with DPBS and transferred to 1.7 mL microfuge tubes. Insoluble protein was pelleted by centrifugation at 16,000 × *g* for 30 min at 4 ˚C. Supernatant containing soluble protein was transferred to tubes or a 96-well plate and aliquots were imaged and blotted as described in "Gel-ABPP with recombinant proteins without immunoprecipitation."

Glutathione (GSH) reactivity assay.

This assay was performed as described previously.[1, 15] Briefly, GSH was diluted to a final concentration of 50 µM 100 mM Tris pH 8.8 with 30% v/v acetonitrile. In triplicate, 100 µL of the GSH solution was added to a clear 384-well plate. 5 µL of 10 mM stereoprobe were then added to the GSH solution to achieve a final probe concentration of 500 µM, and the reaction was incubated to the 2 h and 6 h time points at room temperature. 5 µL of 100 mM Ellman's reagent was then added to the plate and the absorbance was read at 440 nm. The concentration of GSH remaining was derived from a standard curve and the observed rate (k_{obs}/[I]) was calculated assuming pseudo-first-order reaction kinetics from the following equations: $d[GSH]/dt = -k_{obs} \times [GSH]$, $[GSH]_t = [GSH]_{t0} \times e^{-k_{obs}t}$.

Small-molecule docking on ACTMAP

A computationally predicted structure of ACTMAP (AF-Q5BKX5-F1-model_v4), retrieved from the AlphaFold Protein Structure Database,[16-18] was truncated at the N-terminus (aa1-68) due to the low structural organization and low prediction confidence of this protein region (pLDDT < 50). The resulting structure was prepared for calculations (Protein Preparation Workflow). Binding sites were mapped (SiteMap, ≥ 15 points per site, more restrictive definition of hydrophobicity, standard grid). The highest-scoring site (SiteScore 1.072, Dscore 1.004) coincided with the ACTMAP_C132 site and was used as reference to generate a docking grid (Glide Receptor Grid Generation). Ligands (WX-02-623 and WX-02-624) were prepared (LigPrep) and docked on ACTMAP using the previously generated grid (non-covalent, Glide XP, 3 output poses per ligand). Docking output poses were scored (Prime MM-GBSA, VSGB solvation model, OPLS4 force field) and compared based on the scoring output (MM-GBSA dG Bind) as well as the distances from the ACTMAP_C132 sulfur atom to the electrophilic carbon atom in the ligands. All calculations were performed on Schrödinger Maestro version 13.9.138, MMshare version 6.5.138, release 2024-1, platform Darwin-x86_64.

Protein structure figures
Figures showing structures of AGPS and ACTMAP were generated in Chimera-X (version 1.10).[19]

Quantification and data visualization
Band intensities were quantified using Bio-Rad Image Lab software (version 6.1.0). Data visualization in this paper was performed using GraphPad PRISM software version 10.4.2: MS data, public datasets, and band intensities were plotted with mean ± standard deviation (s.d.); IC_{50} curves were generated using a variable slope nonlinear regression setting with the top and bottom constraints set to 100% and 0%, respectively; *P* values were calculated with unpaired two-tailed Student's t-test. Pie charts were generated by Mircrosoft Excel software version 16.76; figures were edited and generated by Adobe Illustrator software version 27.0.1. Workflow for MS treatments and chemical structures were generated by ChemDraw software version 25.0.2.7.

Data availability
The mass spectrometry proteomics data have been deposited to the ProteomeXchange Consortium via the PRIDE[20] partner repository with the dataset identifier PXD074619. Previously published datasets[1] relevant to this study are under data identifier PXD042541. Raw proteomic files were searched using the ProLuCID algorithm using a reverse concatenated, non-redundant variant of the Human UniProt database (release 2016-07). Processed proteomic data are provided in Supporting Dataset 1.

Synthesis and Characterization of Novel Compounds

General considerations

All NMR spectra were recorded at 298 K unless otherwise noted. ^{1}H NMR spectra were recorded on Bruker Avance III 400, Avance III HD 400, or Avance Neo 400 spectrometers (^{1}H, 400 MHz). ^{1}H NMR data are reported as follows: chemical shift (δ), multiplicity (s = singlet, d = doublet, t = triplet, m = multiplet; br = broad, or combinations therein), coupling constants, and integration. Chemical shifts are reported in parts per million (ppm) using the appropriate solvent as reference.[21] Analytical supercritical fluid chromatography (SFC) was performed on a Shimadzu LC system (flow rate: 3 mL/min, back pressure: 100 Bar, column temperature: 35 ºC) equipped with a polydiode array detector. Mass measurements for high-resolution mass spectrometry (HRMS) were performed on a Waters Xevo G2-XS TOF calibrated against sodium formate clusters and using a LeuEnk lockmass. Expected monoisotopic masses were calculated using MassLynx 4.1 and the m/z values for calibrant and lockmass were MassLynx-default values.

Synthesis of novel compounds

All novel compounds were synthesized from previously reported intermediates[1] **S1** and **S2** as described below.

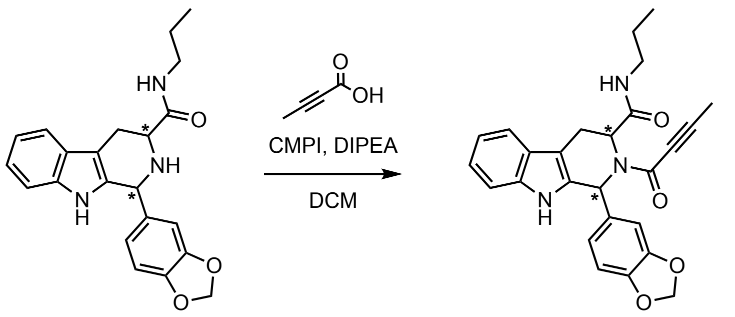

S1 **WX-02-621 – WX-02-624**

Scheme S1: General synthetic scheme for the synthesis of WX-02-621, WX-02-622, WX-02-623, and WX-02-624 from the previously reported intermediates (1*S*,3*S*)-S1, (1*R*,3*R*)-S1, (1*R*,3*S*)-S1, and (1*S*,3*R*)-S1, respectively.

(1*S*,3*S*)-1-(benzo[d][1,3]dioxol-5-yl)-2-(but-2-ynoyl)-*N*-propyl-2,3,4,9-tetrahydro-1*H*-pyrido[3,4-b]indole-3-carboxamide (WX-02-621)

To a precooled (0 ˚C) solution of (1*S*,3*S*)-**S1** (80.0 mg, 191 µmol, 1.0 equiv.) and but-2-ynoic acid (32.0 mg, 382 µmol, 2.0 equiv.) in dichloromethane (DCM, 2 mL) were added *N*,*N*-diisopropylethylamine (DIPEA, 74.0 mg, 572 µmol, 3.0 equiv.) and 2-chloro-1-methylpyridinium iodide (CMPI, 73.0 mg, 286 µmol, 1.5 equiv.) at 0 ˚C. The mixture was warmed to 25 ˚C and stirred for 1 h. Upon reaction completion, the mixture was filtered and concentrated under reduced pressure to give a residue, which was purified by prep-HPLC (column: Waters Xbridge 150mm*25mm*5um; mobile phase: [Waters Xbridge 150mm*25mm*5um; mobile phase: A: water (10 mM NH_4HCO_3), B: acetonitrile; gradient: 42%–72% B over 9 min) to give **WX-02-621** (24.0 mg, 28% yield) as a white solid.

^{1}H NMR (400 MHz, CD_3OD) δ 7.60 – 7.47 (m, 1H), 7.31 – 7.23 (m, 1H), 7.15 – 7.07 (m, 1H), 7.07 – 7.01 (m, 1H), 6.91 – 6.66 (m, 4H), 5.94 – 5.86 (m, 2H), 5.64 – 5.49 (m, 1H), 3.69 (dd, *J* = 15.8, 1.5 Hz, 0.6H), 3.51 (dd, *J* = 15.8, 2.6 Hz, 0.4H), 3.05 – 2.91 (m, 1H), 2.91 – 2.77 (m, 1H), 2.54 – 2.42 (m, 0.4H), 2.36 (ddd, *J* = 13.8, 8.4, 6.3 Hz, 0.6H), 2.18 (s, 1.2H), 2.10 (s, 1.8H), 1.30 – 1.07 (m, 2H), 0.80 – 0.66 (m, 3H), 2 exchangeable protons not observed; 6:4 mixture of rotamers.

HRMS m/z calc. for $C_{26}H_{26}N_3O_4$ $[M+H]^+$ 444.1923 found 444.1934.

(1*R*,3*R*)-1-(benzo[d][1,3]dioxol-5-yl)-2-(but-2-ynoyl)-*N*-propyl-2,3,4,9-tetrahydro-1*H*-pyrido[3,4-b]indole-3-carboxamide (WX-02-622)

To a precooled (0 °C) solution of (1*R*,3*R*)-**S1** (75.0 mg, 199 µmol, 1 equiv.) and but-2-ynoic acid (33.4 mg, 397 µmol, 2.0 equiv.) in DCM (2 mL) were added DIPEA (77.0 mg, 596 µmol, 3.0 equiv.) and CMPI (76.2 mg, 298 µmol, 1.5 equiv.). The mixture was stirred at 25 °C for 1 h. Upon reaction completion, the reaction mixture was concentrated under reduced pressure to give a residue, which was purified by prep-TLC (SiO_2, petroleum ether/EtOAc = 1:1) and prep-HPLC (column: Waters Xbridge 150mm*25mm*5um; mobile phase: A: water (10 mM NH_4HCO_3), B: acetonitrile; gradient: 40%–70% B over 9 min) to give **WX-02-622** (37.0 mg, 42% yield) as an off-white solid.

^{1}H NMR (400 MHz, CD_3OD) δ 7.60 – 7.47 (m, 1H), 7.31 – 7.23 (m, 1H), 7.15 – 7.07 (m, 1H), 7.07 – 7.01 (m, 1H), 6.91 – 6.66 (m, 4H), 5.94 – 5.86 (m, 2H), 5.64 – 5.49 (m, 1H), 3.69 (dd, *J* = 15.8, 1.5 Hz, 0.6H), 3.51 (dd, *J* = 15.8, 2.6 Hz, 0.4H), 3.05 – 2.91 (m, 1H), 2.91 – 2.77 (m, 1H), 2.54 – 2.42 (m, 0.4H), 2.35 (ddd, *J* = 13.1, 8.4, 6.2 Hz, 0.6H), 2.18 (s, 1.2H), 2.09 (s, 1.8H), 1.30 – 1.07 (m, 2H), 0.78 – 0.69 (m, 3H), 2 exchangeable protons not observed; 6:4 mixture of rotamers.

HRMS m/z calc. for $C_{26}H_{26}N_3O_4$ $[M+H]^+$ 444.1923 found 444.1930.

(1*R*,3*S*)-1-(benzo[d][1,3]dioxol-5-yl)-2-(but-2-ynoyl)-*N*-propyl-2,3,4,9-tetrahydro-1*H*-pyrido[3,4-b]indole-3-carboxamide (WX-02-623)

To a solution of (1*R*,3*S*)-**S1** (160 mg, 424 µmol, 1.0 equiv.) and but-2-ynoic acid (40.0 mg, 476 µmol, 1.1 equiv.) in DCM (10 mL) were added DIPEA (164 mg, 1.27 mmol, 3.0 equiv.) and CMPI (162 mg, 636 µmol, 1.5 equiv.). The mixture was stirred at 25 °C for 1 h. Upon reaction completion, the reaction mixture was diluted with water (30 mL) and extracted with DCM (20 mL × 3). The combined organic layers were dried over anhydrous sodium sulfate, filtered and concentrated under reduced pressure to give a residue, which was purified by prep-TLC (SiO_2, EtOAc) and prep-HPLC (column: Waters Xbridge 150mm*25mm*5um; mobile phase: A: water (10 mM NH_4HCO_3), B: acetonitrile; gradient: 38%–68% B over 9 min) to give **WX-02-623** (64.0 mg, 34% yield) as a white solid.

^{1}H NMR (400 MHz, CD_3OD) δ 7.43 – 7.38 (m, 1H), 7.26 (d, *J* = 8.1 Hz, 0.4H), 7.22 (d, *J* = 7.9 Hz, 0.6H), 7.08 – 6.94 (m, 2H), 6.93 – 6.83 (m, 2H), 6.78 (d, *J* = 7.9 Hz, 0.4H), 6.70 (d, *J* = 8.0 Hz, 0.6H), 6.49 (s, 0.4H), 6.18 (s, 0.6H), 5.92 – 5.89 (m, 0.8H), 5.88 – 5.83 (m, 1.2H), 5.54 (dd, *J* = 5.0, 2.8 Hz, 0.6H), 5.14 (dd, *J* = 5.8, 3.8 Hz, 0.4H), 3.59 – 3.37 (m, 2H), 3.07 – 2.98 (m, 2H), 2.02 (s, 1.8H), 1.93 (s, 1.2H), 1.41 – 1.25 (m, 2H), 0.72 (t, *J* = 7.4 Hz, 1.2H), 0.68 (t, *J* = 7.4 Hz, 1.8H), 2 exchangeable protons not observed; 6:4 mixture of rotamers.

HRMS m/z calc. for $C_{26}H_{26}N_3O_4$ $[M+H]^+$ 444.1923 found 444.1931.

(*1S*,3*R*)-1-(benzo[d][1,3]dioxol-5-yl)-2-(but-2-ynoyl)-*N*-propyl-2,3,4,9-tetrahydro-1*H*-pyrido[3,4-b]indole-3-carboxamide (WX-02-624)

To a solution of (1*S*,3*R*)-**S1** (100 mg, 265 µmol, 1.0 equiv.) and but-2-ynoic acid (44.6 mg, 530 µmol, 2.0 equiv.) in DCM (2 mL) were added DIPEA (103 mg, 795 µmol, 3.0 equiv.) and CMPI (102 mg, 397 µmol, 1.5 equiv.). The mixture was stirred at 25 °C for 1 h. Upon reaction completion, the reaction mixture was concentrated under reduced pressure to give a residue, which was purified by prep-TLC (SiO_2, petroleum ether/EtOAc = 2:1) and prep-HPLC (column: Waters Xbridge 150mm*25mm*5um; mobile phase: A: water (10 mM NH_4HCO_3), B: acetonitrile; gradient: 36%–66% B over 9 min) to give **WX-02-624** (38.0 mg, 85.7 µmol, 32% yield) as an off-white solid.

^{1}H NMR (400 MHz, CD_3OD) δ 7.43 – 7.38 (m, 1H), 7.26 (dt, *J* = 8.1, 1.0 Hz, 0.4H), 7.22 (dt, *J* = 8.1, 1.0 Hz, 0.6H), 7.08 – 6.94 (m, 2H), 6.93 – 6.83 (m, 2H), 6.78 (d, *J* = 7.9 Hz, 0.4H), 6.69 (d, *J* = 8.0 Hz, 0.6H), 6.49 (s, 0.4H), 6.18 (s, 0.6H), 5.92 – 5.89 (m, 0.8H), 5.85 (dd, *J* = 5.4, 1.2 Hz, 1.2H), 5.54 (dd, *J* = 5.0, 2.8 Hz, 0.6H), 5.14 (dd, *J* = 5.8, 3.8 Hz, 0.4H), 3.57 – 3.36 (m, 2H), 3.07 – 2.98 (m, 2H), 2.02 (s, 1.8H), 1.92 (s, 1.2H), 1.41 – 1.25 (m, 2H), 0.72 (t, *J* = 7.4 Hz, 1.2H), 0.68 (t, *J* = 7.4 Hz, 1.8H), 2 exchangeable protons not observed; 6:4 mixture of rotamers.

HRMS m/z calc. for $C_{26}H_{26}N_3O_4$ $[M+H]^+$ 444.1923 found 444.1928.

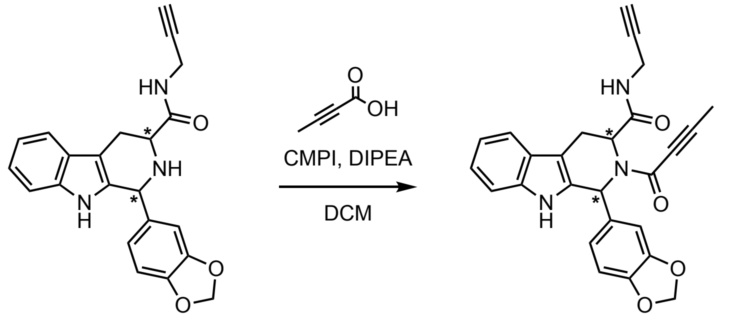

S2 **WX-02-568 – WX-02-571**

Scheme S2: General synthetic scheme for the synthesis of WX-02-568, WX-02-569, WX-02-570, and WX-02-571 from the previously reported intermediates (1*S*,3*S*)-S2, (1*R*,3*R*)-S2, (1*R*,3*S*)-S2, and (1*S*,3*R*)-S2, respectively.

(1*S*,3*S*)-1-(benzo[d][1,3]dioxol-5-yl)-2-(but-2-ynoyl)-*N*-(prop-2-yn-1-yl)-2,3,4,9-tetrahydro-1*H*-pyrido[3,4-b]indole-3-carboxamide (WX-02-568)

To a precooled (0 ºC) solution of (1*S*,3*S*)-**S2** (50.0 mg, 134 µmol, 1.0 equiv.), DIPEA (52.0 mg, 201 µmol, 1.5 equiv.) and CMPI (51.0 mg, 201 µmol, 1.5 equiv.) in DCM (1 mL) was added but-2-ynoic acid (23.0 mg, 268 µmol, 2.0 equiv.). The mixture was warmed to 25 ˚C and stirred for 2 h. Upon reaction completion, the mixture was filtered and concentrated to give a residue, which was purified by prep-HPLC (column: Waters Xbridge C18 150mm*50mm*10um; mobile phase: A: water (10 mM NH_4HCO_3), B: acetonitrile; gradient: 38%–68% B over 10 min) to obtain **WX-02-568** (8.0 mg, 14% yield) as a white solid.

^{1}H NMR (400 MHz, CD_3OD) δ 7.58 – 7.50 (m, 1H), 7.32 – 7.23 (m, 1H), 7.16 – 7.08 (m, 1H), 7.07 – 7.00 (m, 1H), 6.92 – 6.61 (m, 4H), 5.95 – 5.90 (m, 2H), 5.64 – 5.56 (m, 1H), 4.59 (s, 1H), 3.74 – 3.49 (m, 2H), 3.22 – 3.08 (m, 1H), 3.04 – 2.90 (m, 1H), 2.50 – 2.44 (m, 1H), 2.19 (s, 1H), 2.10 (s, 2H), 1 exchangeable proton not observed; 2:1 mixture of rotamers.

HRMS m/z calc. for $C_{26}H_{22}N_3O_4$ $[M+H]^+$ 440.1610 found 440.1618.

(1*R*,3*R*)-1-(benzo[d][1,3]dioxol-5-yl)-2-(but-2-ynoyl)-*N*-(prop-2-yn-1-yl)-2,3,4,9-tetrahydro-1*H*-pyrido[3,4-b]indole-3-carboxamide (WX-02-569)

To a precooled (0 ˚C) solution of (1*R*,3*R*)-**S2** (100.0 mg, 268 µmol, 1.0 equiv.), DIPEA (803 µmol, 140 uL, 3.0 equiv.) and CMPI (103 mg, 402 µmol, 1.5 equiv.) in DCM (2 mL) was added but-2-ynoic acid (45.0 mg, 536 µmol, 2.0 equiv.) at 0 ˚C. The mixture was warmed to 25 ˚C and stirred for 2 h. Upon reaction completion, the reaction mixture was concentrated under reduced pressure to give a residue, which was purified by prep-HPLC (column: Waters Xbridge 150mm*25mm*5um; mobile phase: A: water (10 mM NH_4HCO_3), B: acetonitrile; gradient: 35%–65% B over 8 min) to give **WX-02-569** (37.0 mg, 31% yield) as a white solid.

^{1}H NMR (400 MHz, CD_3OD) δ 7.58 – 7.50 (m, 1H), 7.32 – 7.23 (m, 1H), 7.16 – 7.08 (m, 1H), 7.07 – 7.00 (m, 1H), 6.92 – 6.61 (m, 4H), 5.95 – 5.90 (m, 2H), 5.64 – 5.56 (m, 1H), 4.59 (s, 1H), 3.74 – 3.49 (m, 2H), 3.22 – 3.08 (m, 1H), 3.04 – 2.90 (m, 1H), 2.50 – 2.44 (m, 1H), 2.18 (s, 1H), 2.10 (s, 2H), 1 exchangeable proton not observed; 2:1 mixture of rotamers.

HRMS m/z calc. for $C_{26}H_{22}N_3O_4$ $[M+H]^+$ 440.1610 found 440.1613.

(1*R*,3*S*)-1-(benzo[d][1,3]dioxol-5-yl)-2-(but-2-ynoyl)-*N*-(prop-2-yn-1-yl)-2,3,4,9-tetrahydro-1*H*-pyrido[3,4-b]indole-3-carboxamide (WX-02-570)

To a precooled (0 ˚C) solution of (1*R*,3*S*)-**S2** (125 mg, 335 µmol, 1.0 equiv.), DIPEA (173 mg, 1.3 mmol, 3.9 equiv.) and CMPI (171 mg, 700 µmol, 2.0 equiv.) in DCM (2 mL) was added but-2-ynoic acid (56 mg, 700 µmol, 2.0 equiv.). The mixture was warmed to 25 ˚C and stirred for 2 h. Upon reaction completion, the

mixture was filtered and concentrated to give a residue, which was purified by prep-HPLC (column: Waters Xbridge C18 150mm*50mm*10um; mobile phase: A: water (10 mM NH_4HCO_3), B: acetonitrile; gradient: 34%–64% B over 10 min) to give **WX-02-570** (20.0 mg, 14% yield) as a white solid.
^{1}H NMR (400 MHz, CD_3OD) δ 7.46 – 7.37 (m, 1H), 7.26 (d, *J* = 8.0 Hz, 0.4H), 7.22 (d, *J* = 7.8 Hz, 0.6H), 7.09 – 6.94 (m, 2H), 6.93 – 6.82 (m, 2H), 6.78 (d, *J* = 8.0 Hz, 0.4H), 6.70 (d, *J* = 8.0 Hz, 0.6H), 6.50 (s, 0.4H), 6.19 (s, 0.6H), 5.93 – 5.89 (m, 0.8H), 5.87 – 5.83 (m, 1.2H), 5.53 (t, *J* = 4.0 Hz, 0.6H), 5.09 (dd, *J* = 5.8, 4.3 Hz, 0.4H), 4.59 (s, 1H), 3.94 – 3.73 (m, 2H), 3.49 (d, *J* = 4.1 Hz, 1.2H), 3.42 – 3.23 (m, 0.8H + residual NMR solvent), 2.48 (t, *J* = 2.5 Hz, 0.6H), 2.44 (t, *J* = 2.6 Hz, 0.4H), 2.03 (s, 1.8H), 1.94 (s, 1.2H), 1 exchangeable proton not observed; 6:4 mixture of rotamers.
HRMS m/z calc. for $C_{26}H_{22}N_3O_4$ $[M+H]^+$ 440.1610 found 440.1612.

(1*S*,3*R*)-1-(benzo[d][1,3]dioxol-5-yl)-2-(but-2-ynoyl)-*N*-(prop-2-yn-1-yl)-2,3,4,9-tetrahydro-1*H*-pyrido[3,4-b]indole-3-carboxamide (WX-02-571)
To a precooled (0 ˚C) solution of (1*S*,3*R*)-**S2** (70.0 mg, 187 μmol, 1.0 equiv.), DIPEA (72.7 mg, 0.56 mmol, 3.0 equiv.) and CMPI (71.8 mg, 281 μmol, 1.5 equiv.) in DCM (2 mL) was added but-2-ynoic acid (31.5 mg, 375 μmol, 2.0 equiv.). The mixture was warmed to 25 ˚C and stirred for 2 h. Upon reaction completion, the mixture was filtered and concentrated to give a residue, which was purified by prep-HPLC (column: Waters Xbridge 150mm*25mm* 5um; mobile phase: A: water (10 mM NH_4HCO_3), B: acetonitrile; gradient: 33%–63% B over 8 min) to give **WX-02-571** (20.0 mg, 24% yield) as a white solid.
^{1}H NMR (400 MHz, CD_3OD) δ 7.46 – 7.37 (m, 1H), 7.26 (d, *J* = 8.1 Hz, 0.4H), 7.22 (d, *J* = 8.0 Hz, 0.6H), 7.09 – 6.94 (m, 2H), 6.93 – 6.82 (m, 2H), 6.77 (d, *J* = 8.0 Hz, 0.4H), 6.69 (d, *J* = 8.0 Hz, 0.6H), 6.50 (s, 0.4H), 6.20 (s, 0.6H), 5.93 – 5.89 (m, 0.8H), 5.87 – 5.82 (m, 1.2H), 5.53 (t, *J* = 4.0 Hz, 0.6H), 5.09 (t, *J* = 5.0 Hz, 0.4H), 4.59 (s, 1H, partially exchanged), 3.94 – 3.73 (m, 2H), 3.49 (d, *J* = 4.1 Hz, 1.2H), 3.42 – 3.23 (m, 0.8H + residual NMR solvent), 2.48 (t, *J* = 2.6 Hz, 0.6H), 2.44 (t, *J* = 2.5 Hz, 0.4H), 2.03 (s, 1.8H), 1.93 (s, 1.2H), 1 exchangeable proton not observed; 6:4 mixture of rotamers.
HRMS m/z calc. for $C_{26}H_{22}N_3O_4$ $[M+H]^+$ 440.1610 found 440.1615.

Analytical data: NMR spectra

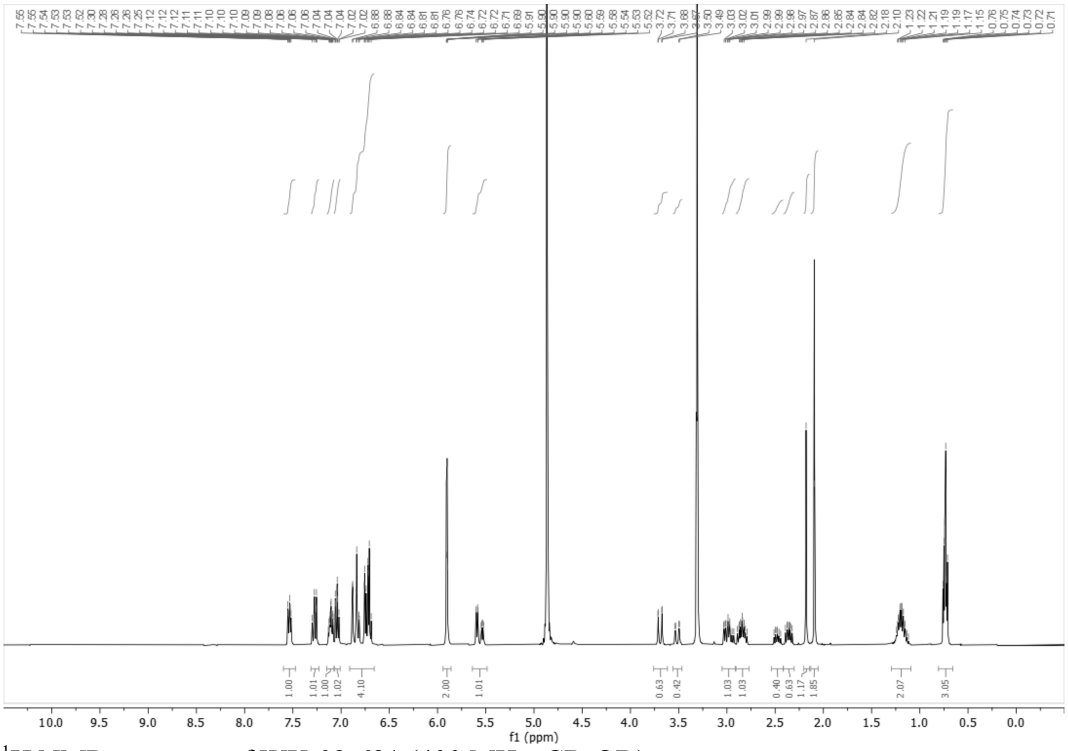

^{1}H NMR spectrum of WX-02-621 (400 MHz, CD_3OD)

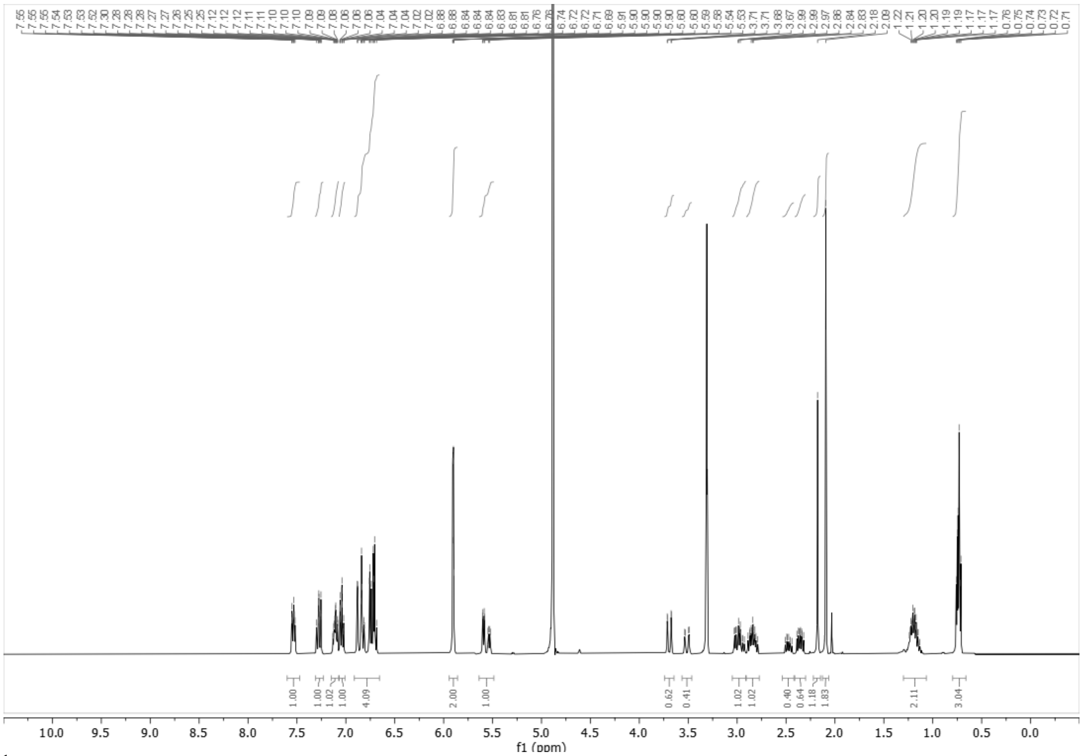

^{1}H NMR spectrum of WX-02-622 (400 MHz, CD_3OD)

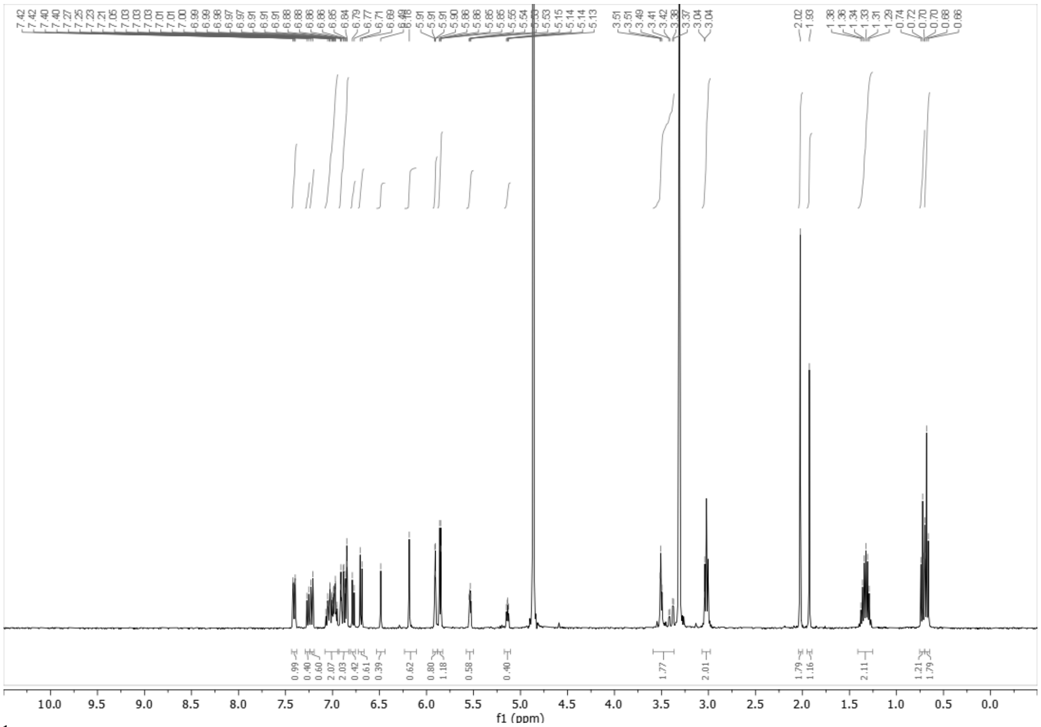

^{1}H NMR spectrum of WX-02-623 (400 MHz, CD_3OD)

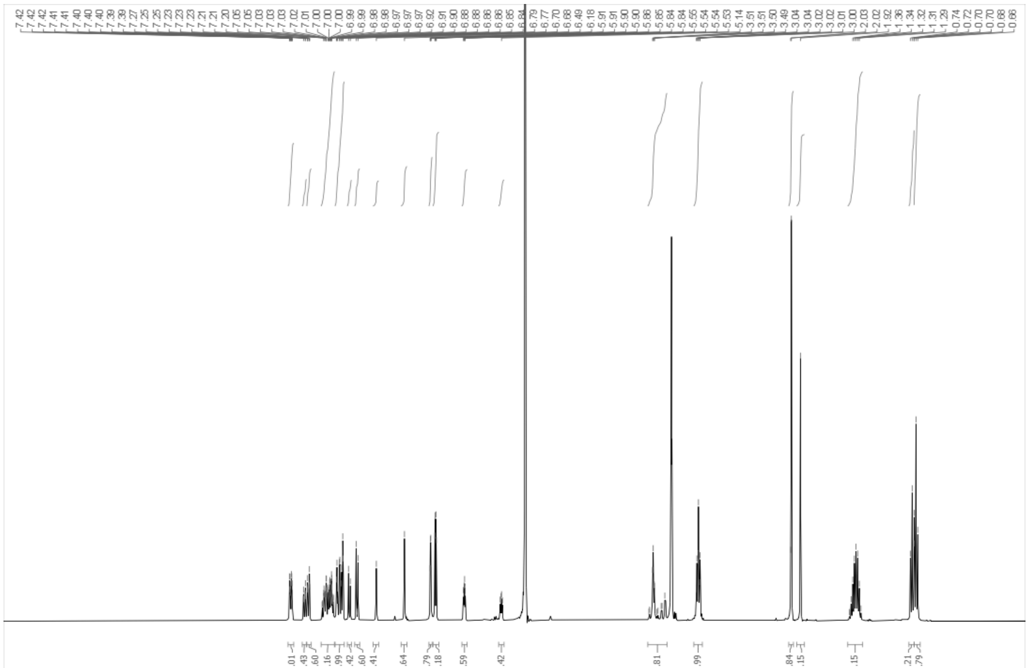

^{1}H NMR spectrum of WX-02-624 (400 MHz, CD_3OD)

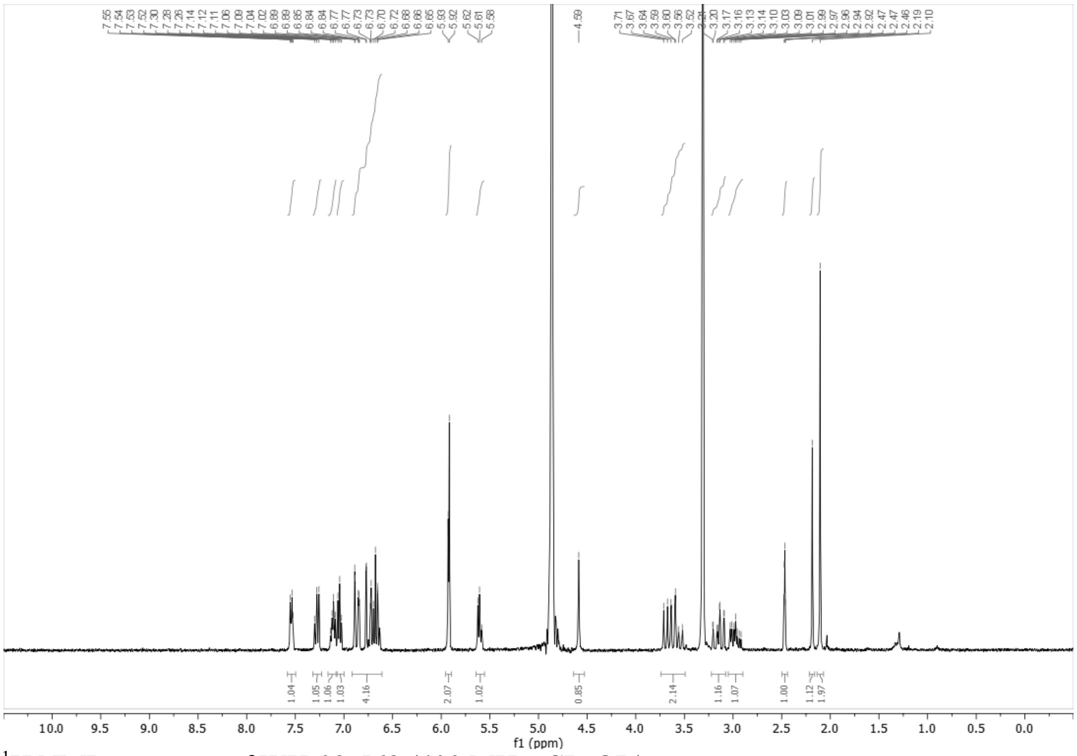

^{1}H NMR spectrum of WX-02-568 (400 MHz, CD_3OD)

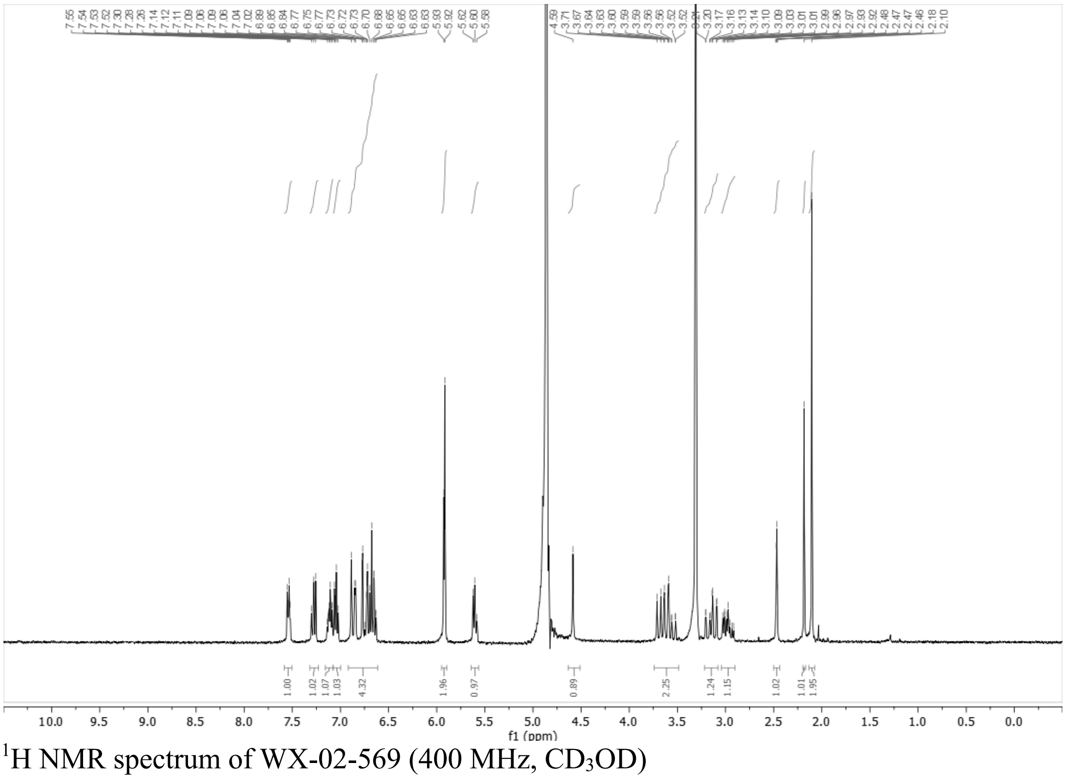

^{1}H NMR spectrum of WX-02-569 (400 MHz, CD_3OD)

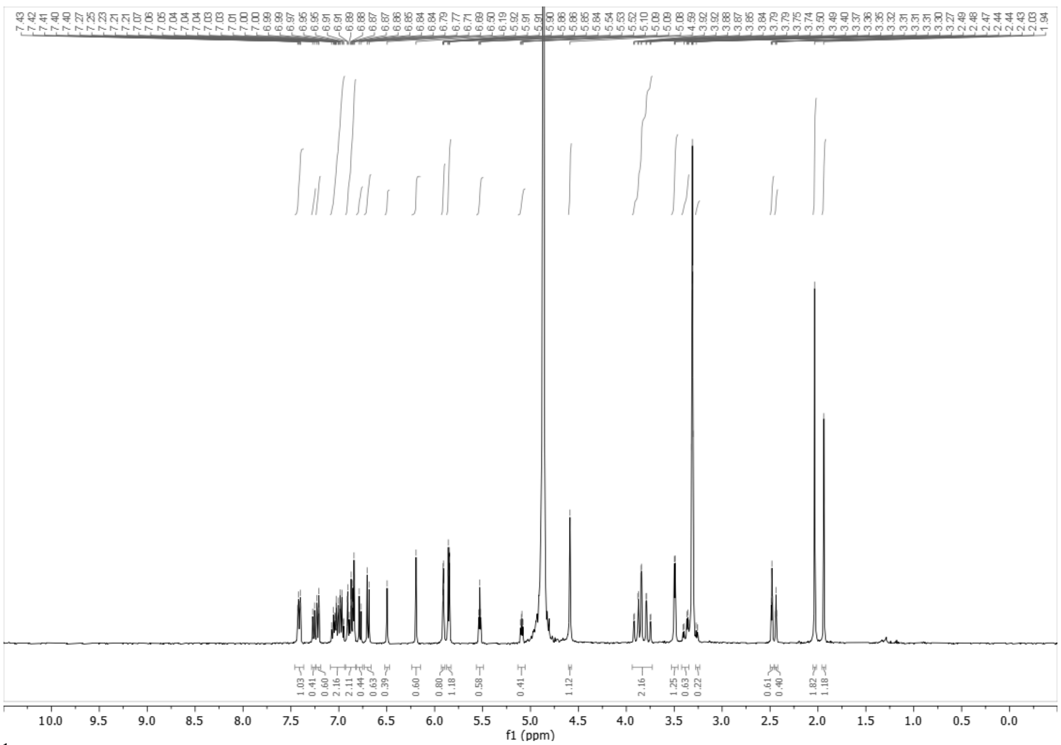

^{1}H NMR spectrum of WX-02-570 (400 MHz, CD_3OD)

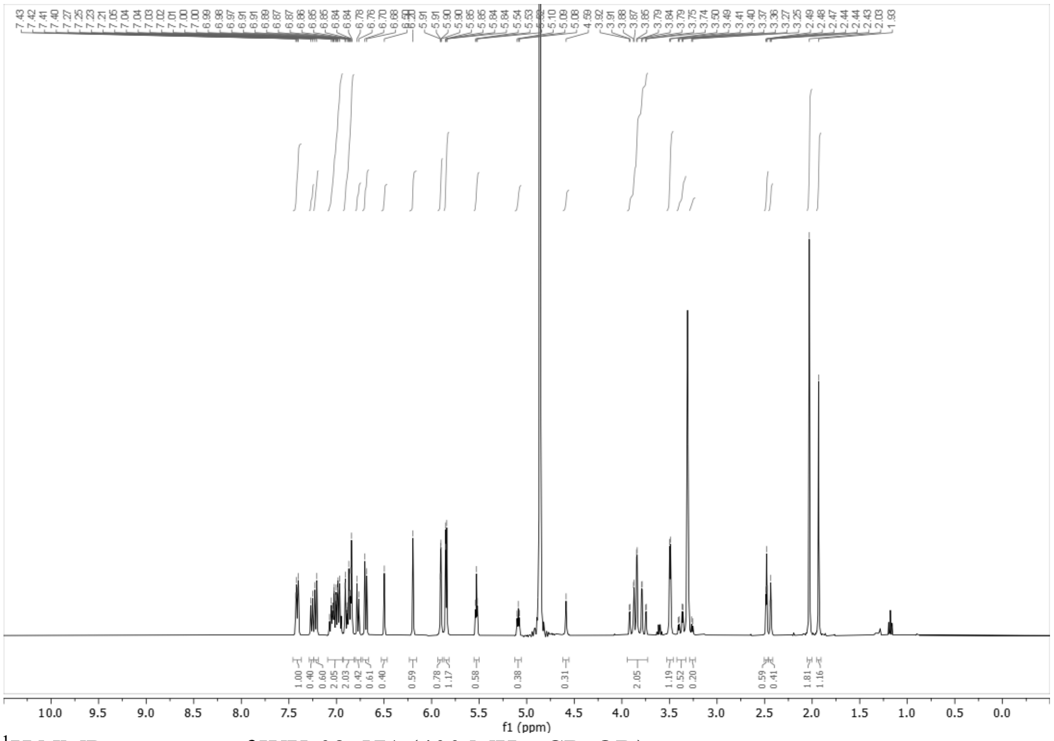

^{1}H NMR spectrum of WX-02-571 (400 MHz, CD_3OD)

Analytical data: SFC

WX-02-621

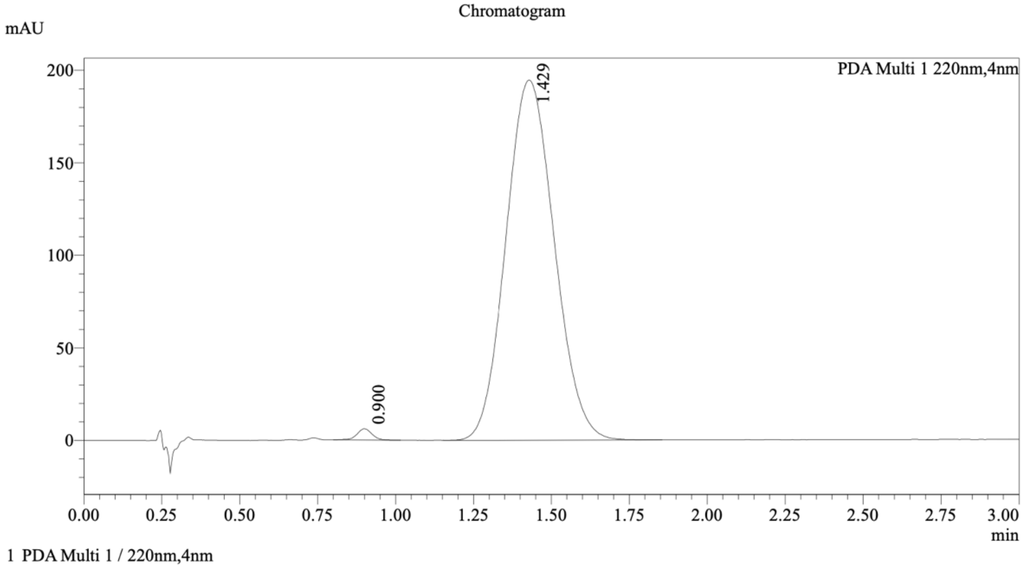

Integration Result

Peak Table

PDA Ch1 220nm

Peak#	Ret. Time	Height	Height%	Resolution(USP)	Area	Area%
1	0.900	6008	2.996	--	19972	0.940
2	1.429	194519	97.004	2.819	2104453	99.060

WX-02-622

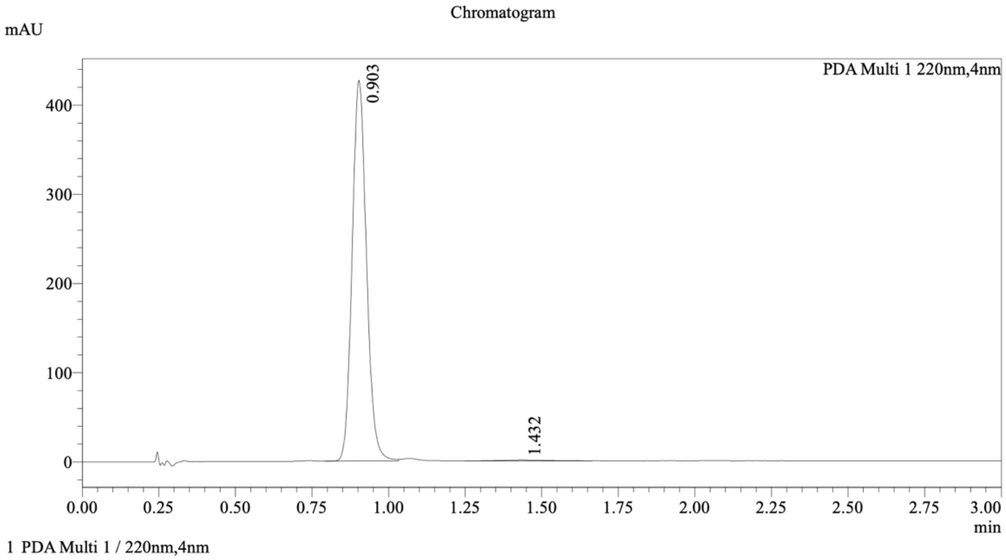

Integration Result

Peak Table

PDA Ch1 220nm

Peak#	Ret. Time	Height	Height%	Resolution(USP)	Area	Area%
1	0.903	423239	99.778	--	1389675	99.312
2	1.432	943	0.222	2.926	9624	0.688

Mixture of WX-02-621 and WX-02-622

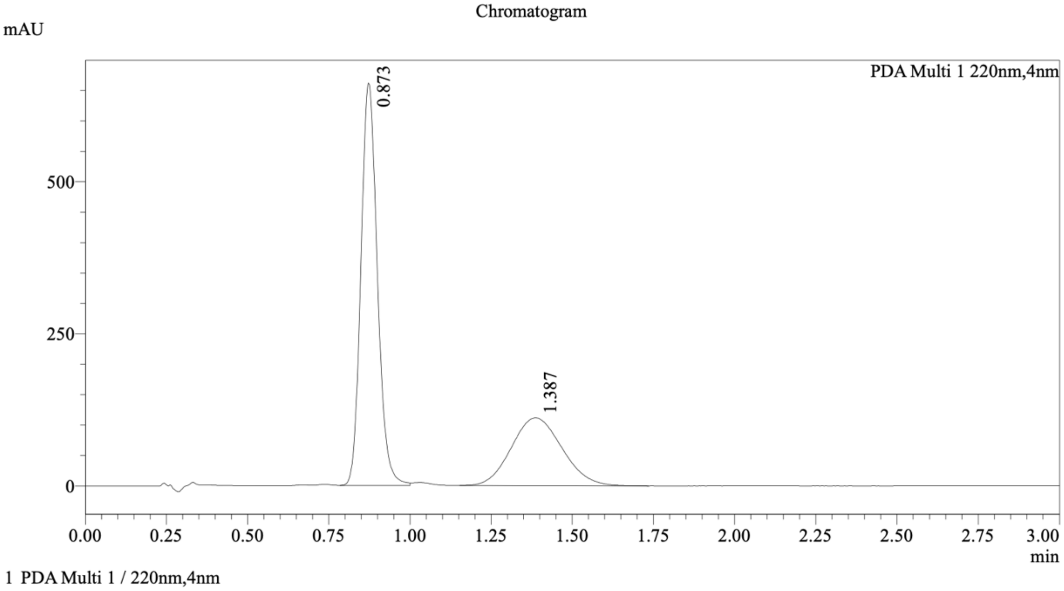

Integration Result

Peak Table

PDA Ch1 220nm

Peak#	Ret. Time	Height	Height%	Resolution(USP)	Area	Area%
1	0.873	651611	85.416	--	2330388	65.743
2	1.387	111260	14.584	2.670	1214286	34.257

Method Details

Column: Chiralpak IG-3 50×4.6mm I.D., 3 µm

Mobile phase: A: CO_2, B: EtOH (0.05% diethylamine)

Elution: 40% B

WX-02-623

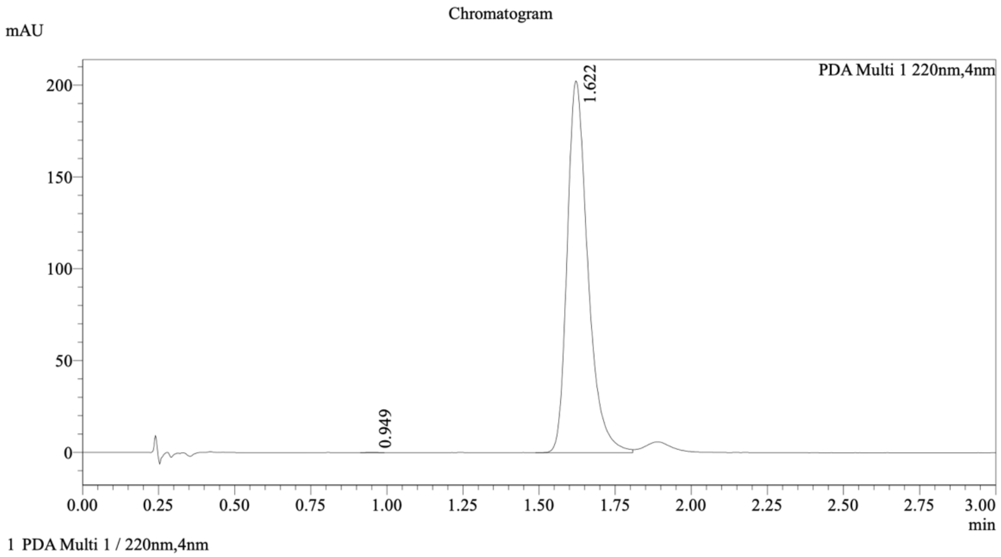

Integration Result

Peak Table

PDA Ch1 220nm

Peak#	Ret. Time	Height	Height%	Resolution(USP)	Area	Area%
1	0.949	126	0.062	--	266	0.028
2	1.622	201816	99.938	7.293	956263	99.972

WX-02-624

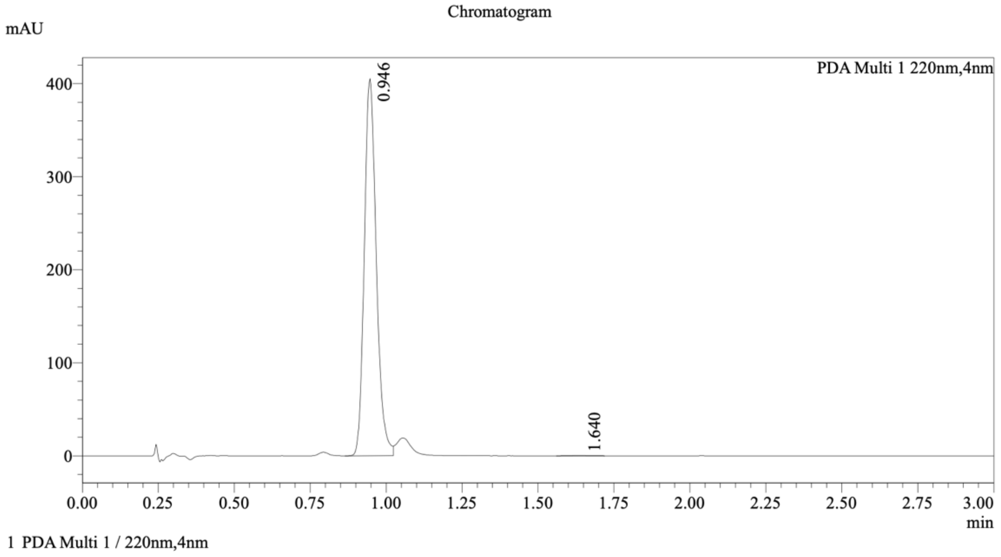

Integration Result

Peak Table

PDA Ch1 220nm

Peak#	Ret. Time	Height	Height%	Resolution(USP)	Area	Area%
1	0.946	395675	99.906	--	1114322	99.855
2	1.640	372	0.094	7.197	1613	0.145

Mixture of WX-02-623 and WX-02-624

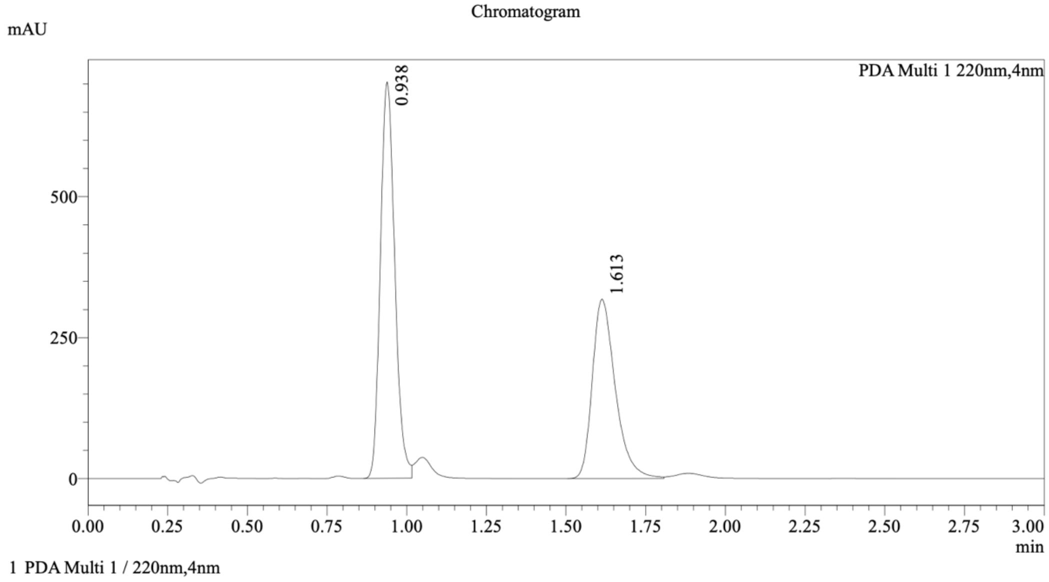

Integration Result

Peak Table

PDA Ch1 220nm

Peak#	Ret. Time	Height	Height%	Resolution(USP)	Area	Area%
1	0.938	691948	68.549	--	2209080	58.013
2	1.613	317479	31.451	6.279	1598812	41.987

Method Details

Column: (S,S)Whelk-O1 50×4.6mm I.D., 3.5 µm
Mobile phase: A: CO_2, B: EtOH (0.05% diethylamine)
Elution: 40% B

WX-02-568

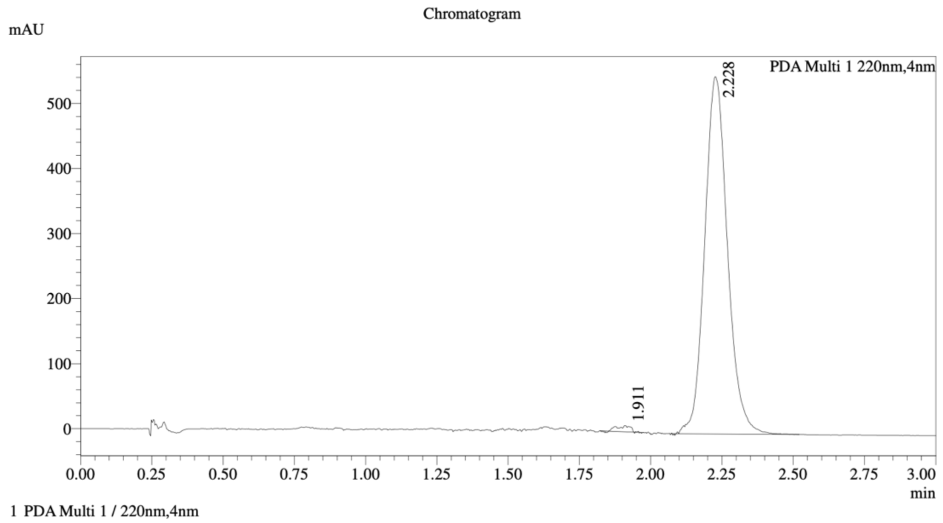

==

Integration Result

==

Peak Table

PDA Ch1 220nm

Peak#	Ret. Time	Height	Height%	Resolution(USP)	Area	Area%
1	1.911	9367	1.683	--	30758	0.997
2	2.228	547359	98.317	3.140	3054527	99.003

WX-02-569

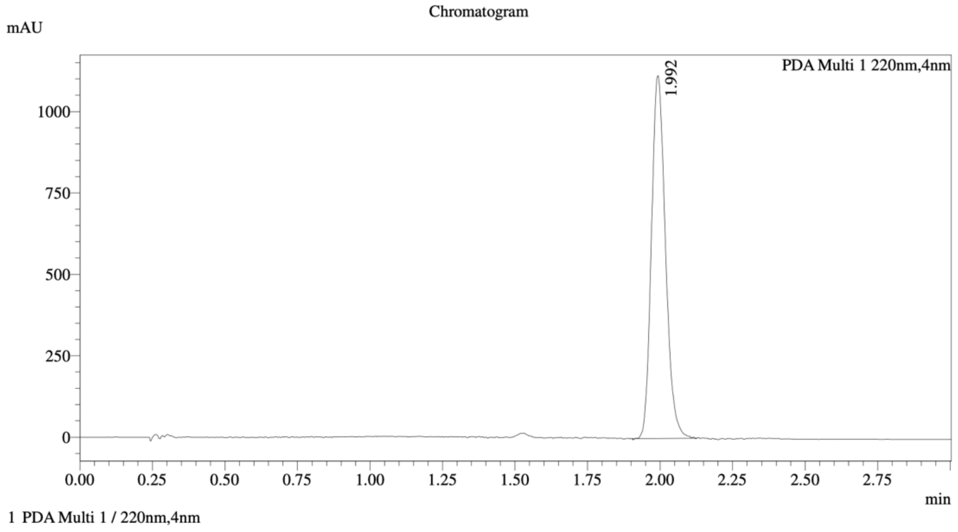

==

Integration Result

==

Peak Table

PDA Ch1 220nm

Peak#	Ret. Time	Height	Height%	Resolution(USP)	Area	Area%
1	1.992	1098053	100.000	--	3755129	100.000

Mixture of WX-02-568 and WX-02-569

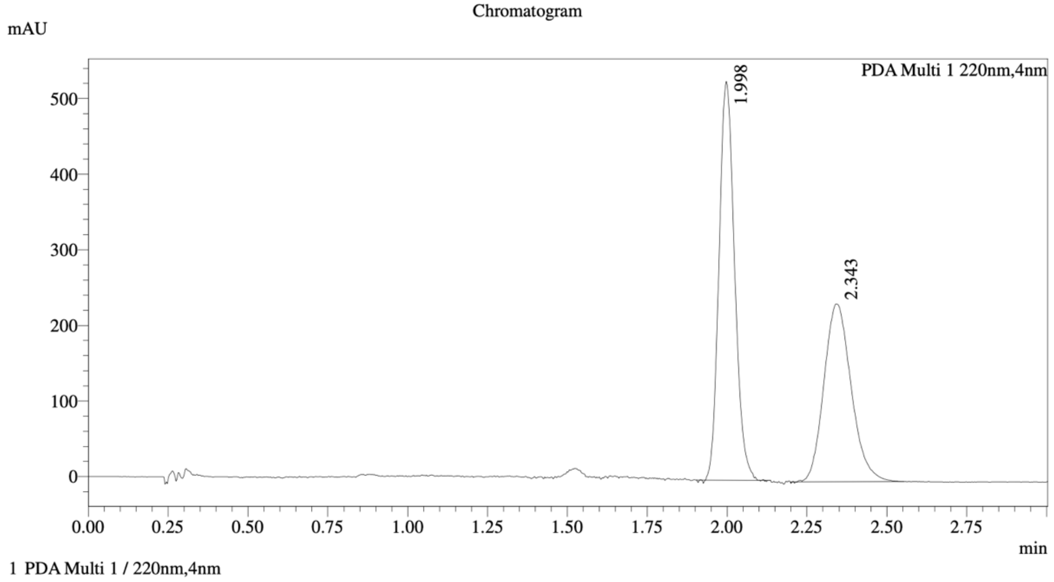

==

Integration Result

==

Peak Table

PDA Ch1 220nm

Peak#	Ret. Time	Height	Height%	Resolution(USP)	Area	Area%
1	1.998	524064	69.024	--	1785090	55.985
2	2.343	235189	30.976	2.776	1403426	44.015

Method Details

Column: Chiralcel OJ-3 50×4.6mm I.D., 3 µm
Mobile phase: A: CO_2, B: MeOH (0.05% diethylamine)
Elution: 5% to 40% B (gradient)

WX-02-570

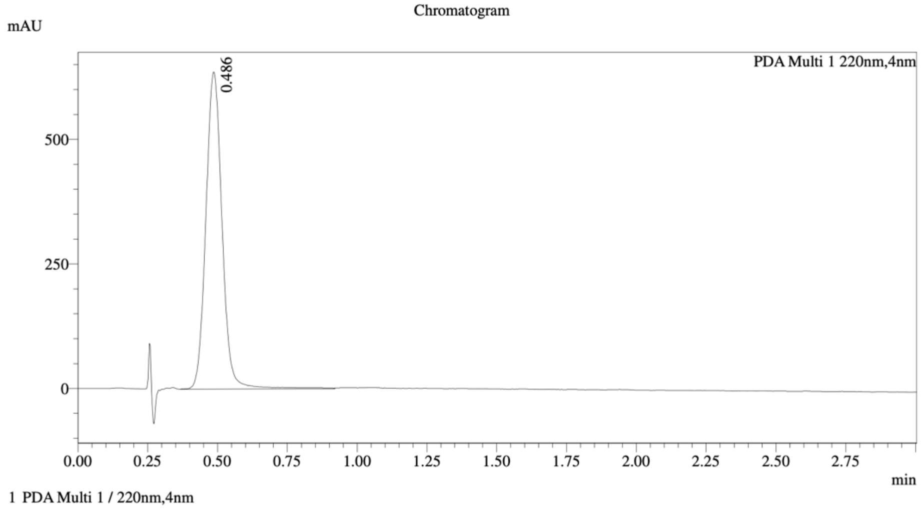

==

Integration Result

==

Peak Table

PDA Ch1 220nm

Peak#	Ret. Time	Height	Height%	Resolution(USP)	Area	Area%
1	0.486	634232	100.000	--	2573390	100.000

WX-02-571

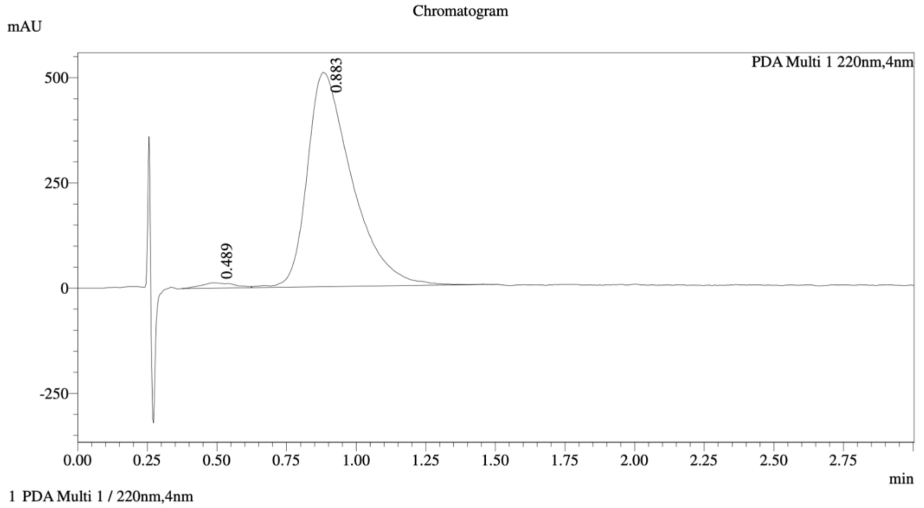

==

Integration Result

==

Peak Table

PDA Ch1 220nm

Peak#	Ret. Time	Height	Height%	Resolution(USP)	Area	Area%
1	0.489	12459	2.393	--	102502	1.771
2	0.883	508169	97.607	1.587	5686077	98.229

Mixture of WX-02-570 and WX-02-571

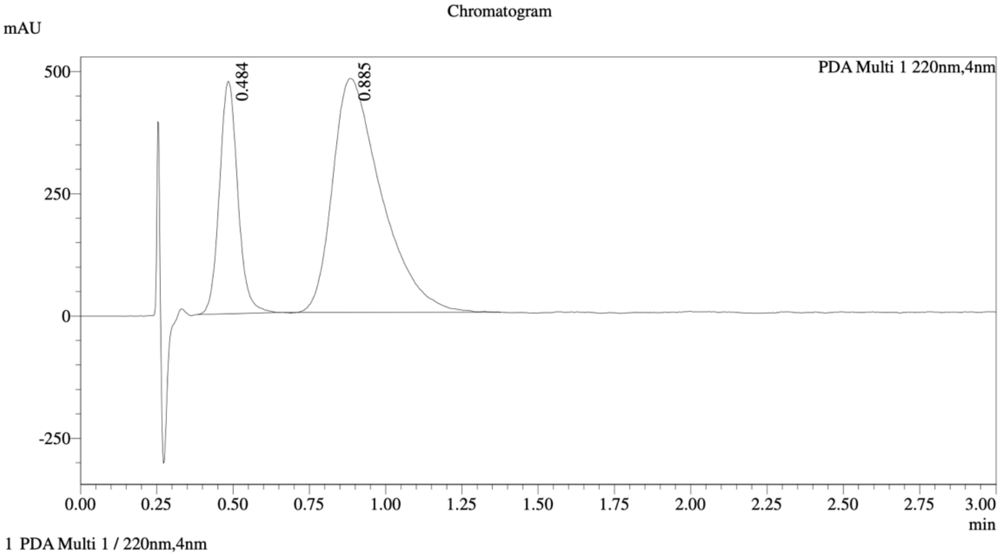

==
Integration Result
==

Peak Table

PDA Ch1 220nm

Peak#	Ret. Time	Height	Height%	Resolution(USP)	Area	Area%
1	0.484	473932	49.775	--	1987842	27.133
2	0.885	478224	50.225	2.003	5338577	72.867

Method Details

Column: Chiralcel OD-3 50×4.6mm I.D., 3 µm
Mobile phase: A: CO_2, B: 2:1 MeOH/acetonitrile (0.05% diethylamine)
Elution: 40% B

References

(1) Njomen, E.; Hayward, R. E.; DeMeester, K. E.; Ogasawara, D.; Dix, M. M.; Nguyen, T.; Ashby, P.; Simon, G. M.; Schreiber, S. L.; Melillo, B.; Cravatt, B. F. Multi-tiered chemical proteomic maps of tryptoline acrylamide-protein interactions in cancer cells. *Nat. Chem.* **2024**, *16* (10), 1592-1604.
(2) Rostovtsev, V. V.; Green, L. G.; Fokin, V. V.; Sharpless, K. B. A stepwise huisgen cycloaddition process: copper(I)-catalyzed regioselective "ligation" of azides and terminal alkynes. *Angew. Chem. Int. Ed.* **2002**, *41* (14), 2596-2599.
(3) Tornoe, C. W.; Christensen, C.; Meldal, M. Peptidotriazoles on solid phase: [1,2,3]-triazoles by regiospecific copper(i)-catalyzed 1,3-dipolar cycloadditions of terminal alkynes to azides. *J. Org. Chem.* **2002**, *67* (9), 3057-3064.
(4) Speers, A. E.; Cravatt, B. F. Profiling enzyme activities in vivo using click chemistry methods. *Chem Biol* **2004**, *11* (4), 535-546.
(5) Lazear, M. R.; Remsberg, J. R.; Jaeger, M. G.; Rothamel, K.; Her, H. L.; DeMeester, K. E.; Njomen, E.; Hogg, S. J.; Rahman, J.; Whitby, L. R.; Won, S. J.; Schafroth, M. A.; Ogasawara, D.; Yokoyama, M.; Lindsey, G. L.; Li, H.; Germain, J.; Barbas, S.; Vaughan, J.; Hanigan, T. W.; Vartabedian, V. F.; Reinhardt, C. J.; Dix, M. M.; Koo, S. J.; Heo, I.; Teijaro, J. R.; Simon, G. M.; Ghosh, B.; Abdel-Wahab, O.; Ahn, K.; Saghatelian, A.; Melillo, B.; Schreiber, S. L.; Yeo, G. W.; Cravatt, B. F. Proteomic discovery of chemical probes that perturb protein complexes in human cells. *Mol. Cell* **2023**, *83* (10), 1725-1742 e1712.
(6) Vinogradova, E. V.; Zhang, X.; Remillard, D.; Lazar, D. C.; Suciu, R. M.; Wang, Y.; Bianco, G.; Yamashita, Y.; Crowley, V. M.; Schafroth, M. A.; Yokoyama, M.; Konrad, D. B.; Lum, K. M.; Simon, G. M.; Kemper, E. K.; Lazear, M. R.; Yin, S.; Blewett, M. M.; Dix, M. M.; Nguyen, N.; Shokhirev, M. N.; Chin, E. N.; Lairson, L. L.; Melillo, B.; Schreiber, S. L.; Forli, S.; Teijaro, J. R.; Cravatt, B. F. An Activity-Guided Map of Electrophile-Cysteine Interactions in Primary Human T Cells. *Cell* **2020**, *182* (4), 1009-1026 e1029.
(7) Arafeh, R.; Shibue, T.; Dempster, J. M.; Hahn, W. C.; Vazquez, F. The present and future of the Cancer Dependency Map. *Nat. Rev. Cancer* **2025**, *25* (1), 59-73.
(8) Haahr, P.; Galli, R. A.; van den Hengel, L. G.; Bleijerveld, O. B.; Kazokaite-Adomaitiene, J.; Song, J. Y.; Kroese, L. J.; Krimpenfort, P.; Baltissen, M. P.; Vermeulen, M.; Ottenheijm, C. A. C.; Brummelkamp, T. R. Actin maturation requires the ACTMAP/C19orf54 protease. *Science* **2022**, *377* (6614), 1533-1537.
(9) Li, H.; Ma, T.; Remsberg, J. R.; Won, S. J.; DeMeester, K. E.; Njomen, E.; Ogasawara, D.; Zhao, K. T.; Huang, T. P.; Lu, B.; Simon, G. M.; Melillo, B.; Schreiber, S. L.; Lykke-Andersen, J.; Liu, D. R.; Cravatt, B. F. Assigning functionality to cysteines by base editing of cancer dependency genes. *Nat. Chem. Biol.* **2023**, *19* (11), 1320-1330.
(10) Hayward, R. E.; Berkeley, R. F.; Gao, Z.; Garhammer, M.; Morizono, M. A.; Njomen, E.; Li, H.; DeMeester, K. E.; Cociorva, V.; Herzik, M. A.; Ulrich Hartl, F.; Melillo, B.; Cravatt, B. F. Tryptoline Stereoprobe Elaboration Identifies Inhibitors of the GRPEL1-HSPA9 Chaperone Complex. *bioRxiv* **2025**, 2025.2010.2020.683548. DOI: 10.1101/2025.10.20.683548
(11) Goetzke, F. W.; Bernard, S. M.; Ju, C. W.; Pollock, J.; DeMeester, K. E.; Gross, J.; Simon, G. M.; He, C.; Melillo, B.; Cravatt, B. F. Complexoform-restricted covalent TRMT112 ligands that allosterically agonize METTL5. *Nat Chem Biol* **2026**, *22*, 770-782.
(12) Kavanagh, M. E.; Horning, B. D.; Khattri, R.; Roy, N.; Lu, J. P.; Whitby, L. R.; Ye, E.; Brannon, J. C.; Parker, A.; Chick, J. M.; Eissler, C. L.; Wong, A. J.; Rodriguez, J. L.; Rodiles, S.; Masuda, K.; Teijaro, J. R.; Simon, G. M.; Patricelli, M. P.; Cravatt, B. F. Selective inhibitors of

JAK1 targeting an isoform-restricted allosteric cysteine. *Nat. Chem. Biol.* **2022**, *18* (12), 1388-1398.
(13) Baltgalvis, K. A.; Lamb, K. N.; Symons, K. T.; Wu, C. C.; Hoffman, M. A.; Snead, A. N.; Song, X.; Glaza, T.; Kikuchi, S.; Green, J. C.; Rogness, D. C.; Lam, B.; Rodriguez-Aguirre, M. E.; Woody, D. R.; Eissler, C. L.; Rodiles, S.; Negron, S. M.; Bernard, S. M.; Tran, E.; Pollock, J.; Tabatabaei, A.; Contreras, V.; Williams, H. N.; Pastuszka, M. K.; Sigler, J. J.; Pettazzoni, P.; Rudolph, M. G.; Classen, M.; Brugger, D.; Claiborne, C.; Plancher, J. M.; Cuartas, I.; Seoane, J.; Burgess, L. E.; Abraham, R. T.; Weinstein, D. S.; Simon, G. M.; Patricelli, M. P.; Kinsella, T. M. Chemoproteomic discovery of a covalent allosteric inhibitor of WRN helicase. *Nature* **2024**, *629* (8011), 435-442.
(14) Shalem, O.; Sanjana, N. E.; Hartenian, E.; Shi, X.; Scott, D. A.; Mikkelson, T.; Heckl, D.; Ebert, B. L.; Root, D. E.; Doench, J. G.; Zhang, F. Genome-scale CRISPR-Cas9 knockout screening in human cells. *Science* **2014**, *343* (6166), 84-87.
(15) Liu, Z.; Remsberg, J. R.; Li, H.; Njomen, E.; DeMeester, K. E.; Tao, Y.; Xia, G.; Hayward, R. E.; Yoo, M.; Nguyen, T.; Simon, G. M.; Schreiber, S. L.; Melillo, B.; Cravatt, B. F. Proteomic Ligandability Maps of Spirocycle Acrylamide Stereoprobes Identify Covalent ERCC3 Degraders. *J Am Chem Soc* **2024**, *146* (15), 10393-10406.
(16) Jumper, J.; Evans, R.; Pritzel, A.; Green, T.; Figurnov, M.; Ronneberger, O.; Tunyasuvunakool, K.; Bates, R.; Žídek, A.; Potapenko, A.; Bridgland, A.; Meyer, C.; Kohl, S. A. A.; Ballard, A. J.; Cowie, A.; Romera-Paredes, B.; Nikolov, S.; Jain, R.; Adler, J.; Back, T.; Petersen, S.; Reiman, D.; Clancy, E.; Zielinski, M.; Steinegger, M.; Pacholska, M.; Berghammer, T.; Bodenstein, S.; Silver, D.; Vinyals, O.; Senior, A. W.; Kavukcuoglu, K.; Kohli, P.; Hassabis, D.; Jumper, J.; Evans, R.; Pritzel, A.; Green, T.; Figurnov, M.; Ronneberger, O.; Tunyasuvunakool, K.; Bates, R.; Žídek, A.; Potapenko, A.; Bridgland, A.; Meyer, C.; Kohl, S. A. A.; Ballard, A. J.; Cowie, A.; Romera-Paredes, B.; Nikolov, S.; Jain, R.; Adler, J.; Back, T.; Petersen, S.; Reiman, D.; Clancy, E.; Zielinski, M.; Steinegger, M.; Pacholska, M.; Berghammer, T.; Bodenstein, S.; Silver, D.; Vinyals, O.; Senior, A. W.; Kavukcuoglu, K.; Kohli, P.; Hassabis, D. Highly accurate protein structure prediction with AlphaFold. *Nature* **2021**, *596* (7873), 583-589.
(17) Varadi, M.; Anyango, S.; Deshpande, M.; Nair, S.; Natassia, C.; Yordanova, G.; Yuan, D.; Stroe, O.; Wood, G.; Laydon, A.; Zidek, A.; Green, T.; Tunyasuvunakool, K.; Petersen, S.; Jumper, J.; Clancy, E.; Green, R.; Vora, A.; Lutfi, M.; Figurnov, M.; Cowie, A.; Hobbs, N.; Kohli, P.; Kleywegt, G.; Birney, E.; Hassabis, D.; Velankar, S. AlphaFold Protein Structure Database: massively expanding the structural coverage of protein-sequence space with high-accuracy models. *Nucleic Acids Res.* **2022**, *50* (D1), D439-D444.
(18) Varadi, M.; Bertoni, D.; Magana, P.; Paramval, U.; Pidruchna, I.; Radhakrishnan, M.; Tsenkov, M.; Nair, S.; Mirdita, M.; Yeo, J.; Kovalevskiy, O.; Tunyasuvunakool, K.; Laydon, A.; Žídek, A.; Tomlinson, H.; Hariharan, D.; Abrahamson, J.; Green, T.; Jumper, J.; Birney, E.; Steinegger, M.; Hassabis, D.; Velankar, S. AlphaFold Protein Structure Database in 2024: providing structure coverage for over 214 million protein sequences. *Nucleic Acids Res.* **2024**, *52* (D1), 368-378.
(19) Goddard, T. D.; Huang, C. C.; Meng, E. C.; Pettersen, E. F.; Couch, G. S.; Morris, J. H.; Ferrin, T. E. UCSF ChimeraX: Meeting modern challenges in visualization and analysis. *Protein Sci.* **2018**, *27* (1), 14-25.

(20) Perez-Riverol, Y.; Bandla, C.; Kundu, D. J.; Kamatchinathan, S.; Bai, J.; Hewapathirana, S.; John, N. S.; Prakash, A.; Walzer, M.; Wang, S.; Vizcaino, J. A. The PRIDE database at 20 years: 2025 update. *Nucleic Acids Res* **2025**, *53* (D1), D543-D553.
(21) Fulmer, G. R.; Miller, A. J. M.; Sherden, N. H.; Gottlieb, H. E.; Nudelman, A.; Stoltz, B. M.; Bercaw, J. E.; Goldberg, K. I. NMR Chemical Shifts of Trace Impurities: Common Laboratory Solvents, Organics, and Gases in Deuterated Solvents Relevant to the Organometallic Chemist. *Organometallics* **2010**, *29* (9), 2176-2179.
